# NAT10 mediated ac4C acetylation driven m^6^A modification via involvement of YTHDC1-LDHA/PFKM regulates glycolysis and promotes osteosarcoma

**DOI:** 10.1186/s12964-023-01321-y

**Published:** 2024-01-17

**Authors:** Zhongting Mei, Zhihua Shen, Jiaying Pu, Qian Liu, Guoxin Liu, Xuting He, Yang Wang, Jinrui Yue, Shiyu Ge, Tao Li, Ye Yuan, Lei Yang

**Affiliations:** 1 Department of Orthopedics, The First Affiliated Hospital of Harbin Medical University, Harbin, China; 2 Department of Pharmacology, (The State-Province Key Laboratories of Biomedicine Pharmaceutics of China, Key Laboratory of Cardiovascular Research, Ministry of Education), College of Pharmacy, Harbin Medical University, Harbin, China; 3 Department of Clinical Pharmacology, College of Pharmacy, Harbin Medical University, Harbin, China; 4 National Key Laboratory of Frigid Cardiovascular Disease, Harbin, China; 5 Department of Pharmacy, (The University Key Laboratory of Drug Research, Heilongjiang Province), The Second Affiliated Hospital of Harbin Medical University, Harbin, China; 6 Key Laboratory of Hepatosplenic Surgery of Ministry of Education, The First Affiliated Hospital of Harbin Medical University, Harbin, China; 7 NHC Key Laboratory of Cell Transplantation, The First Affiliated Hospital of Harbin Medical University, Harbin, China

**Keywords:** Osteosarcoma, ac4C acetylation, m^6^A methylation, NAT10, Glycolysis, YTHDC1, PFKM, LDHA

## Abstract

The dynamic changes of RNA N6-methyladenosine (m^6^A) during cancer progression participate in various cellular processes. However, less is known about a possible direct connection between upstream regulator and m^6^A modification, and therefore affects oncogenic progression. Here, we have identified that a key enzyme in N4-acetylcytidine (ac4C) acetylation NAT10 is highly expressed in human osteosarcoma tissues, and its knockdown enhanced m^6^A contents and significantly suppressed osteosarcoma cell growth, migration and invasion. Further results revealed that NAT10 silence inhibits mRNA stability and translation of m^6^A reader protein YTHDC1, and displayed an increase in glucose uptake, a decrease in lactate production and pyruvate content. YTHDC1 recognizes differential m^6^A sites on key enzymes of glycolysis phosphofructokinase (PFKM) and lactate dehydrogenase A (LDHA) mRNAs, which suppress glycolysis pathway by increasing mRNA stability of them in an m^6^A methylation-dependent manner. YTHDC1 partially abrogated the inhibitory effect caused by NAT10 knockdown in tumor models in vivo, lentiviral overexpression of YTHDC1 partially restored the reduced stability of YTHDC1 caused by lentiviral depleting NAT10 at the cellular level. Altogether, we found ac4C driven RNA m^6^A modification can positively regulate the glycolysis of cancer cells and reveals a previously unrecognized signaling axis of NAT10/ac4C-YTHDC1/m^6^A-LDHA/PFKM in osteosarcoma.

Video Abstract

**Supplementary Information:**

The online version contains supplementary material available at 10.1186/s12964-023-01321-y.

## Introduction

Osteosarcoma is the most prevalent primary bone malignancy tumor, characterized by a high degree of malignancy and an unfavorable prognosis. The 5-year survival rate following metastasis is less than 20% [1, 2]. The disease typically occurs in weight-bearing long bones, most commonly in the distal femur and proximal tibia, and accounts for about three-quarters of all osteosarcomas [1, 3]. Thus, exploration of the molecular mechanism underlying the development and progression of osteosarcoma is imperative for developing novel therapeutic strategies. Altered energy metabolism is a biochemical fingerprint of cancer cells that represents one of the ‘‘hallmarks of cancer”. This metabolic phenotype is characterized by preferential reliance on glycolysis, which involves the conversion of glucose into pyruvate followed by lactate production for energy generation in an oxygen-independent manner [4]. Targeting the glycolysis pathway has been considered as novel therapeutic strategy for cancer treatment.

RNA methylation modification is reported to account for more than 60% of all RNA modifications, and N6-methyladenosine (m^6^ A) is the most common modification on higher biological mRNAs and lncRNAs [5–7]. In humans, it widely occurs in numerous processes including precursor mRNA (pre-mRNA) splicing, mRNA translation, stability and structure maintenance, exportation and decay [5, 6]. Recent studies have demonstrated that the m^6^A demethylases were dysregulated in several malignant tumors [8–10]. Our studie has demonstrated that the down-regulation of the demethylase ALKBH5 in osteosarcoma cells/tissues was associated with an increased m^6^A methylation compared with normal osteoblasts/tissues. Overexpression of ALKBH5 significantly inhibited the growth, migration, invasion and apoptosis of osteosarcoma cells [11].

N4-acetylcytidine (ac4C) is a conservative chemical modification of tRNA, rRNA and mRNA. This unique modification occurs exclusively in eukaryotic RNA, with all characteristic sites of ac4C located at the central nucleotide of the 5'-CCG-3' consensus sequence [12–14]. N-acetyltransferase 10 (NAT10) is the sole human enzyme known to possess both acetyltransferase and RNA-binding activities. NAT10 has been linked to cancer and premature aging syndromes, emphasizing its potential importance in human disease pathology [15, 16]. Ac4C has the unique ability to modulate RNA–protein interactions and provides the basis for fine-tuning the properties of nucleic acid therapy through N4-cytidineacylation [17]. Recent studies have demonstrated that NAT10 plays a pivotal role in the development of colon cancer by affecting the stability of FSP1 mRNA and ferroptosis, underscoring its potential as a new prognostic and therapeutic target for colon cancer [18]. Given the known crucial role of NAT10 in various types of tumors, there has been an intense focus on characterizing the effects of this enzyme on the development and progression of osteosarcoma.

Herein, we found that ac4C driven RNA m^6^ A modification can positively regulate the glycolysis of cancer cells. NAT10-dependent ac4C acetylation regulates mRNA stability and translation of YTHDC1. YTHDC1 (YTH N6-Methyladenosine RNA Binding Protein C1) is a protein coding gene and binds N6-methyladenosine(m^6^A)-containing RNAs. YTHDC1 recognizes differential m^6^A sites on PFKM and LDHA RNAs, which promotes osteosarcoma cell growth and regulates glycolysis pathway by increasing PFKM and LDHA mRNA stability in an m^6^A methylation-dependent manner. Consistent with these results, YTHDC1 partially abrogated the inhibitory effect of NAT10 knockdown in tumor models in vivo. Together, these findings demonstrate a mechanism of ac4C driven RNA m^6^A modification and reveals a previously unrecognized signaling axis of NAT10-YTHDC1-LDHA/PFKM in osteosarcoma, shedding new light on the epigenetic regulation in human cancer.

## Results

### Ac4C regulates mRNA stability and translation of YTHDC1

**Fig. 1:**
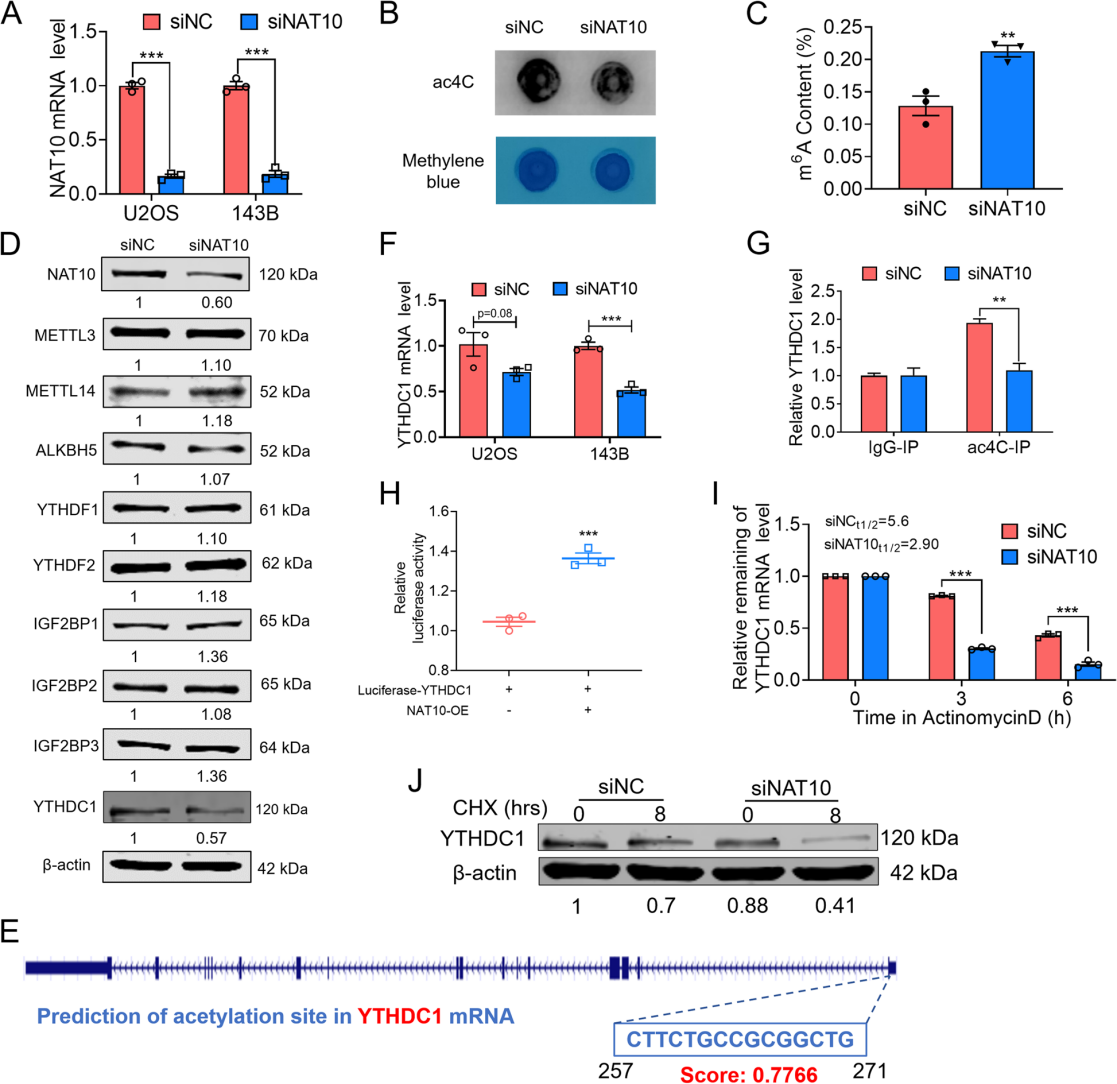
Ac4C regulates mRNA stability and translation of YTHDC1. **A** U2OS and 143B cells were transfected with negative control (NC) siRNA or NAT10 siRNA for 24 h. The knockdown efficiency of NAT10 siRNA in U2OS and 143B cells was evaluated by qRT-PCR analysis. **B** The specificity of the ac4C antibody was detected in 143B cells by Dot blot analyses. **C** The m^6^A content in 143B cells was detected by ELISA assay. **D** Protein expression of NAT10, METTL3, METTL14, ALKBH5, YTHDF1, YTHDF2, IGF2BP1, IGF2BP2, IGF2BP3, YTHDC1 in 143B cells was detected by western blotting. **E** Prediction diagram of YTHDC1 mRNA acetylation site. **F** qRT-PCR analysis revealed the expression of YTHDC1 after knockdown of NAT10 in U2OS and 143B cells. **G** RIP-qPCR assay was performed to analyze the mRNA levels of ac4C-modified in 143B cells with or without knockdown of NAT10. **H** Luciferase activity assay was performed to confirm the YTHDC1 mRNA was directly bound to NAT10 in HEK-293 T cells. **I** The YTHDC1 mRNA half-life was estimated according to linear regression analysis after the indicated actinomycin D treatment. **J** 143B cells were transfected with negative control (NC) siRNA or NAT10 siRNA for 24 h and then treated with cycloheximide (CHX)-mediated for 8 h. Western blot analysis of YTHDC1 protein levels over time in response to DNA damage. Data are expressed as mean ± SEM. **P < 0.01; ***P < 0.001

Our previous studies have found that m^6^A contents were significantly increased in osteosarcoma cells [11]. We firstly clarified whether ac4C could regulate the m^6^A methylation in osteosarcoma. It has been reported that NAT10 is the key enzyme for ac4C modification of mRNA [19]. As depicted in Fig. 1A, we confirmed the transfection efficiency of NAT10 siRNA by both qRT-PCR in U2OS and 143B cells. RNA dot blot revealed that ac4C content was significantly decreased in osteosarcoma cells after transfected with NAT10 siRNA (Fig. 1B). And the change of m^6^A content was exhibited by Elisa assay, the m^6^A content was significantly increased after knockdown NAT10 as compared with the NC group (Fig. 1C). Therefore, NAT10-dependent ac4C acetylation regulates the m^6^A methylation levels of osteosarcoma cells. To identify specific targets responsible for NAT10-induced osteosarcoma cells m^6^A methylation. The influence of knockdown NAT10 upon the expression of genes associated with these m^6^A ‘‘writer’’ (METTL3, METTL14), ‘‘erasers’’ (ALKBH5) and ‘‘readers’’ (YTHDF1, YTHDF2, IGF2BP1, IGF2BP2, IGF2BP3, YTHDC1) were detected in 143B cells (Fig. 1D). Specifically, m^6^A writers IGF2BP1 and IGF2BP3 were significantly increased by more than 20% in siNAT10-treated 143B cells. In contrast, only the expression of YTHDC1 was significantly reduced after knocking down NAT10 (Fig. 1D), and the change of YTHDC1 expression was inconsistent with the change trend of m^6^A modification. Next, we predicted the ac4C acetylation sites in YTHDC1 mRNA with PACES (http://www.rnanut.net/paces/), as shown in Fig. 1E. The mRNA expression level of YTHDC1 was detected by qRT-PCR in U2OS and 143B osteosarcoma cell lines after NAT10 knocking down (Fig. 1F). Furthermore, we performed gene-specific ac4C qPCR for YTHDC1 and confirmed the reduction of ac4C levels in YTHDC1 transcripts, demonstrating the reliability of our predicted site (Fig. 1G). To further confirm that YTHDC1 is a direct target gene of NAT10, we co-transfected HEK-293 T cells with NAT10 overexpression plasmid and YTHDC1 luciferase vectors. Dual-luciferase reporter assay showed that the luciferase activity elicited by the vector containing mouse wild type (WT) YTHDC1 was promoted by overexpressing NAT10 (Fig. 1H). Thus, we hypothesized that YTHDC1 transcripts are potential targets of ac4C acetylation. To further explore the potential mechanism of NAT10 regulating YTHDC1 transcripts expression, we found that siNAT10 inhibited the stability of YTHDC1 mRNA in the presence of transcription inhibitor actinomycin D (Act D) as compared with the siNC group in 143B cells (Fig. 1I). Meanwhile, siNAT10 accelerated the degradation of YTHDC1 in the presence of translation inhibitor cycloheximide (CHX) (Fig. 1J), and NAT10 silencing indeed resulted in a significant decrease in the half-life of YTHDC1 transcripts (from 5.6 to 2.9 h).

### NAT10-dependent ac4C acetylation impacts the growth and motility of osteosarcoma cells

**Fig. 2:**
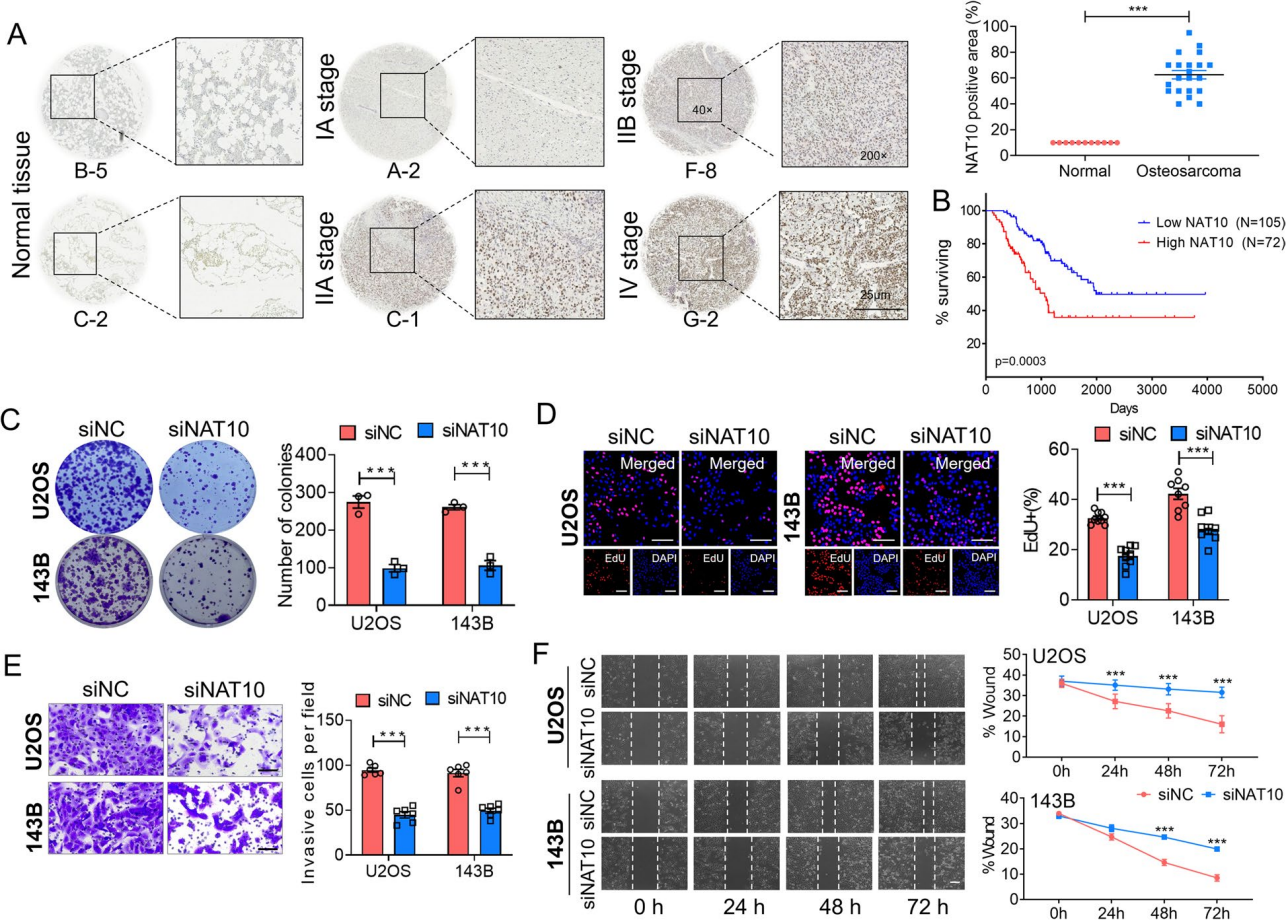
NAT10-dependent ac4C acetylation impacts the growth and motility of osteosarcoma cells. **A** Immunohistochemistry (IHC) analysis of NAT10 protein expression on tissue microarrays (TMAs) composed of benign bone tissues tissues (*n* = 20), IIA stage osteosarcomas (*n* = 31), IV stage osteosarcomas (*n* = 31) tumor cores. Representative IHC images (magnification 40 and 200) are presented (Bar: 25 μm). **B** Kaplan–Meier analysis using data derived from the The Cancer Genome Atlas (TCGA) cohort revealed that low expression of the NAT10 was associated with a good prognosis in patients with sarcoma. **C** Representative images and quantitative results of cancer cell colonies. **D** Cell proliferation was measured using an EdU staining kit (Bar: 40 μm). **E** Cell invasion was detected by transwell assay (Bar: 100 μm); **F** Migration ability was determined by wound-healing assay at 0, 24, 48 and 72 h, respectively (Bar: 100 μm). Data are expressed as mean ± SEM. ***P < 0.001

We then applied immunohistochemistry (IHC) assays to measure NAT10 protein expression in an osteosarcoma tissue microarray (TMA) containing 71 tissue cores as compared with normal bone tissues (Supplementary Fig. 1). Significantly higher NAT10 protein expression was detected in osteosarcoma cores compared with normal bone tissues (Fig. 2A). Kaplan–Meier survival analysis from the The Cancer Genome Atlas (TCGA) dataset http://www.oncolnc.org/) showed that sarcoma patients with low NAT10 expression exhibited a superior survival, while sarcoma patients with high NAT10 expression exhibited poor survival rate (Fig. 2B). To determine whether NAT10 regulated ac4C acetylation plays a role in osteosarcoma cells, we conducted loss-of-function studies. We examined the effect of NAT10 on cell proliferation, migration and invasion. The results showed that depleting NAT10 decreased the colony-formation capacity of U2OS and 143B osteosarcoma cells (Fig. 2C). Furthermore, NAT10 knockdown remarkably inhibited cell proliferation, invasion and migration abilities in both U2OS and 143B cells in EdU staining (Fig. 2D), transwell cell invasion (Fig. 2E) and wound healing cell migration (Fig. 2F) assays. These results demonstrate that NAT10 affects cell proliferation, migration and angiogenesis in vitro.

### Ac4C acetylation regulates glycolysis of osteosarcoma cells

**Fig. 3:**
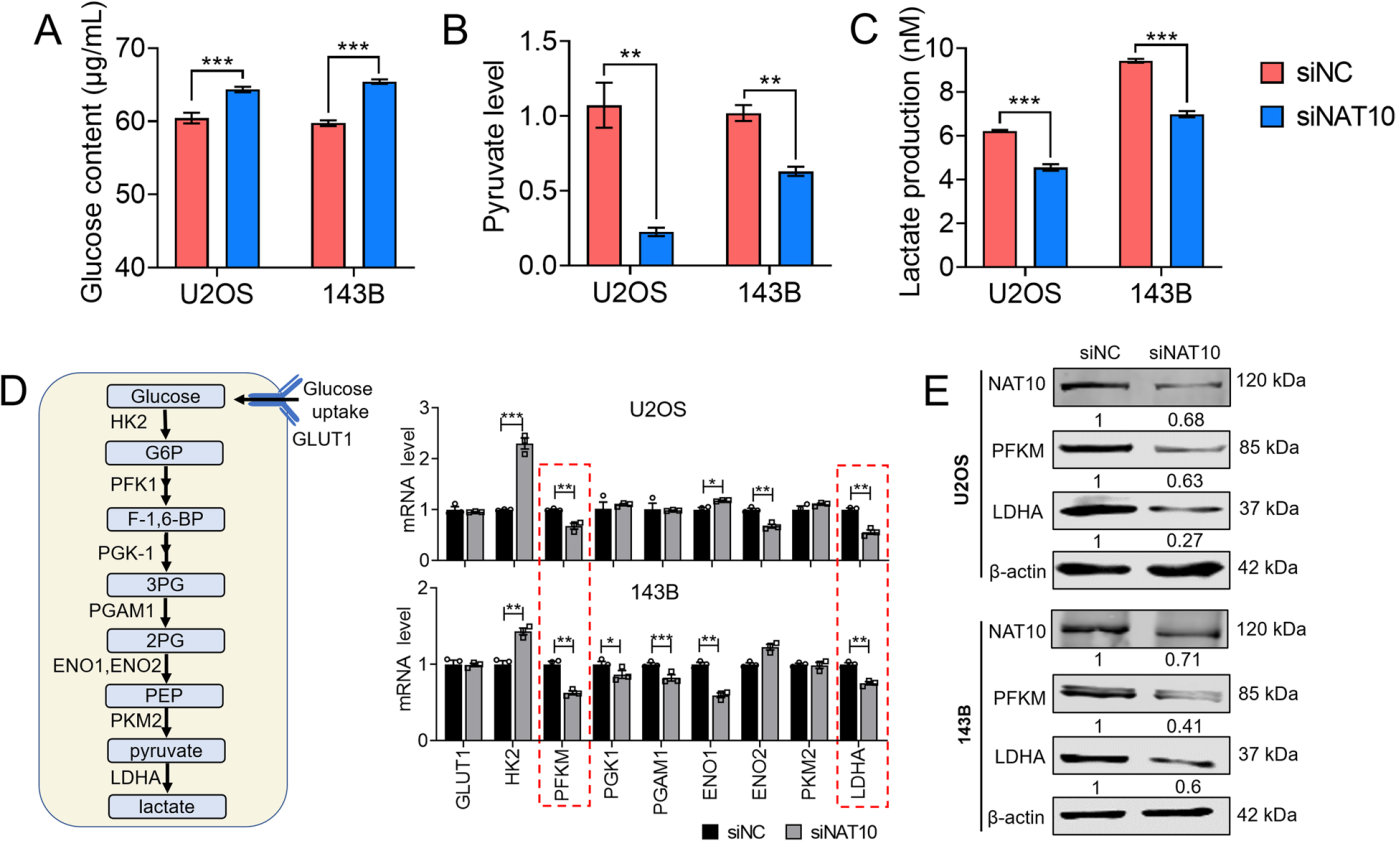
ac4C acetylation regulates glycolysis of osteosarcoma cells. **A** U2OS and 143B cells were transfected with NC siRNA or NAT10 siRNA for 24 h. Glucose content was measured by a glucose uptake colorimetric assay. **B** Pryuvate level was measured by a pyruvate uptake assay. **C** Lactate production in cells was measured by a lactate colorimetric assay. **D** Schematic diagram of key enzymes of glycolytic pathway and mRNA levels of related genes in U2OS and 143B cell lines. **E** Protein expression of NAT10, PFKM and LDHA in U2OS and 143B cells transfected with NC or NAT10 siRNA detected by western blotting. Data are expressed as mean ± SEM. *P < 0.05; **P < 0.01; ***P < 0.001

Metabolic reprogramming is of great significance in the progression of various cancers, with the glycolysis pathway being a well-established metabolic process that drives cancer progression. Metabolisms are also altered in malignant osteosarcoma cells. Among reprogrammed metabolisms, alterations in glycolysis are essential to the massive biosynthesis and energy demands of osteosarcoma cells to sustain their growth and metastasis [20]. Next, we investigated the potential mechanism of NAT10 in regulating glycolysis in osteosarcoma cells. After NAT10 knockdown in two cell lines, we measured the differences in glucose, lactate, and pyruvate content in cells or culture medium. lines. As expected, knockdown of NAT10 in 143B and U2OS cells resulted in an increase in glucose content (Fig. 3A), and an overall decrease in lactate production, and pyruvate content as compared with siNC group (Fig. 3B, C). Hypoxia has been found to enhance lactate production and tumor growth through activation of HIF1-α, GLUT1, HK2, PKM2, PDK, ENO1 or LDHA. LDHA is a key enzyme in the final step of glycolysis, catalyzing the conversion of pyruvate to lactic acid, which in turn promotes Warburg effect and tumor growth. PFK1, the first rate-limiting enzyme in the glycolytic pathway, is a key metabolic hub in glycolysis regulation, PFKM is an isoform of PFK1 [21]. The qRT-PCR results showed that in 143B and U2OS cells, the mRNA levels of glycolytic related genes, especially PFKM and LDHA, were decreased to varying degrees after knockdown of NAT10 (Fig. 3D). We subsequently tested the change of PFKM and LDH at the protein level and got the same results (Fig. 3E). These findings indicate that NAT10, as a key enzyme in ac4C acetylation, plays a role in mediating glycolysis in osteosarcoma cells.

### YTHDC1 mediated m^6^A methylation impacts osteosarcoma cell growth and regulates glycolysis pathway

**Fig. 4:**
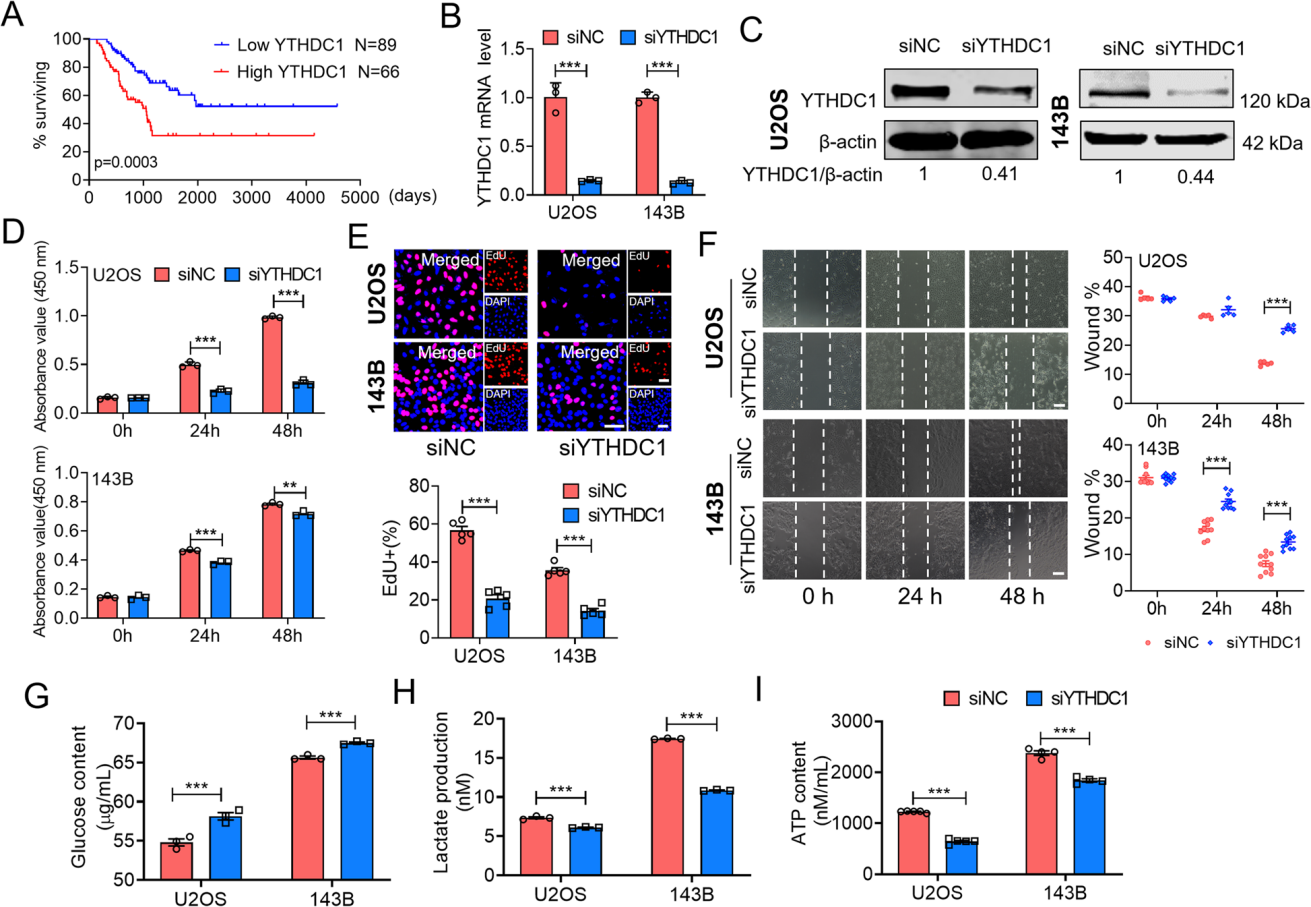
YTHDC1 mediated m^6^A methylation impacts osteosarcoma cell growth and regulates glycolysis pathway. **A** Kaplan–Meier survival curve indicates the difference in survival rate between YTHDC1 high expression and YTHDC1 low expression patients. **B**, **C** U2OS and 143B cells were transfected with NC siRNA or YTHDC1 siRNA for 24 h. The knockdown efficiency of YTHDC1 siRNA in U2OS and 143B cells was evaluated by qRT-PCR analysis and western blot analysis. **D** Cell viability was detected by CCK8 assay at 0, 24 and 48 h (Bar: 100 μm). **E** Effect of YTHDC1 knockdown on proliferation of U2OS and 143B cells. **F** Migration ability was detected by wound-healing assay at 0 h, 24 h and 48 h respectively with YTHDC1 silencing in U2OS and 143B cells. **G, H** YTHDC1 was knocked down in 143B and U2OS cell lines, then glucose content and lactate production were detected. **I** ATP content was measured by an ATP assay. Data are expressed as mean ± SEM. ***P < 0.001

YTHDC1 is a nuclear m^6^A reader, that plays a critical role in tumor development by a m^6^A-dependent manner [20]. As a key enzyme in ac4C acetylation, NAT10 can regulate the stability of YTHDC1. Therefore, we hypothesized that NAT10 could affect glycolysis through YTHDC1. Increased glycolysis is mainly caused by elevated expression of enzymes and transporters involved in glucose uptake, lactate production and lactate secretion. Kaplan–Meier analysis using data derived from the TCGA cohort also revealed that low expression of the YTHDC1 was associated with a good prognosis in patients with sarcoma (Fig. 4A). We first, verified the knockdown efficiency of YTHDC1 at mRNA and protein levels (Fig. 4B, C). In addition, we found that knocking down YTHDC1 inhibited osteosarcoma cells growth (Fig. 4D), proliferation (Fig. 4E) and migration (Fig. 4F) as compared with siNC group in U2OS and 143B cells. Interestingly, after knocking down YTHDC1, the residual glucose in the cell culture medium and the contents of lactate and pyruvate in the cells were measured, and the results are in line with expectations. That is to say knockdown of YTHDC1 in 143B and U2OS cells resulted in an overall decrease in lactate production and pyruvate content as compared with these in the siNC group (Fig. 4H, I). Glucose uptake levels were upregulated after YTHDC1 knockdown in two cell lines (Fig. 4G). Therefore, these findings indicate that NAT10 affects osteosarcoma cell growth and regulates glycolysis pathways through YTHDC1-mediated m^6^A methylation.

### YTHDC1 recognizes differential m^6^A on PFKM and LDHA RNAs

**Fig. 5:**
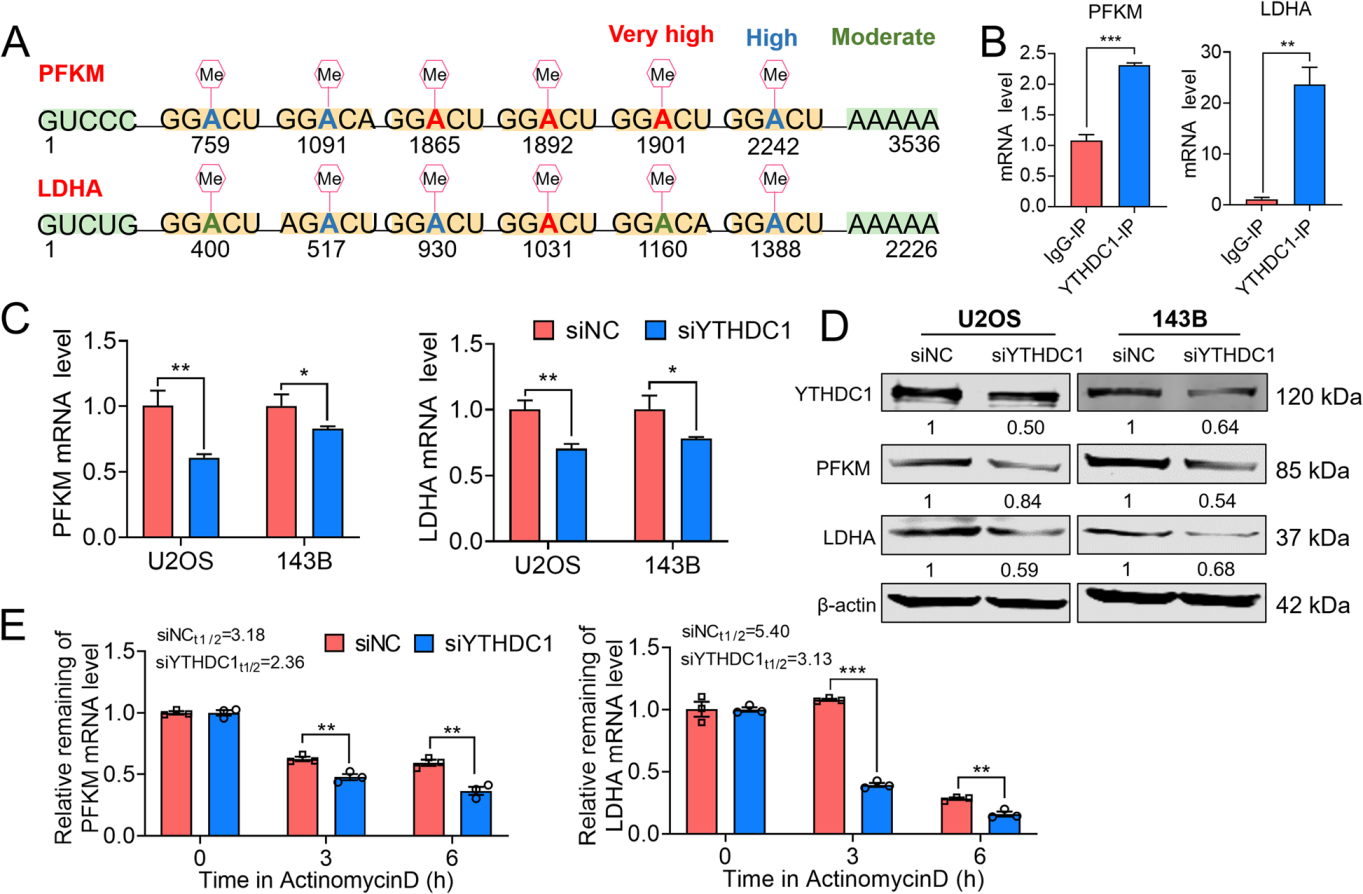
YTHDC1 recognizes differential m^6^A on PFKM and LDHA RNAs. **A** Schematic representation of m^6^A modification sites on mRNA of PFKM and LDHA. **B** The colocalization of YTHDC1 with PFK1M and LDHA was confirmed by RIP-qPCR. **C**, **D** The expression level of YTHDC1, PFK1M and LDHA were verified by qRT-PCR analysis and Western blotting in YTHDC1 knockdown cell lines. **E** The PFKM and LDHA mRNA half-lives were estimated according to linear regression analysis after the indicated actinomycin D treatment. Data are expressed as mean ± SEM. *P < 0.05; **P < 0.01; ***P < 0.001

Next, we went on to get further insight into the mechanisms accounting for our findings. According to a sequence-based SRAMP m^6^A modification site predictor (http://www.cuilab.cn/sramp), we observed mRNA of PFKM and LDHA genes carrying several potential m^6^A modification sites, as shown in Fig. 5A. The results of RIP-PCR assay verified that PFKM and LDHA could bind to YTHDC1 specifically (Fig. 5B). In addition, we found that YTHDC1 could affect the expression of PFKM and LDHA at both mRNA and protein levels in 143B and U2OS cell lines (Fig. 5C, D). To further investigate the underlying mechanism by which YTHDC1 regulates PFKM and LDHA expression, we treated NC or YTHDC1 knockdown 143B cells with actinomycin D (Act D) and actinomycin CHX, a protein synthesis inhibitor and determined the half-life and mRNA stability of PFKM and LDHA transcripts. Notably, YTHDC1 silencing did result in significant reductions in the half-lives of both transcripts (3.18–2.36 h, 5.40–3.13 h, Fig. 5E). These data indicate that YTHDC1 recognizes differential m^6^A on PFKM and LDHA RNAs, which affects their stability and translation efficiency.

### YTHDC1 partially abrogated the inhibitory effect of NAT10 knockdown on tumor growth in vivo

**Fig. 6:**
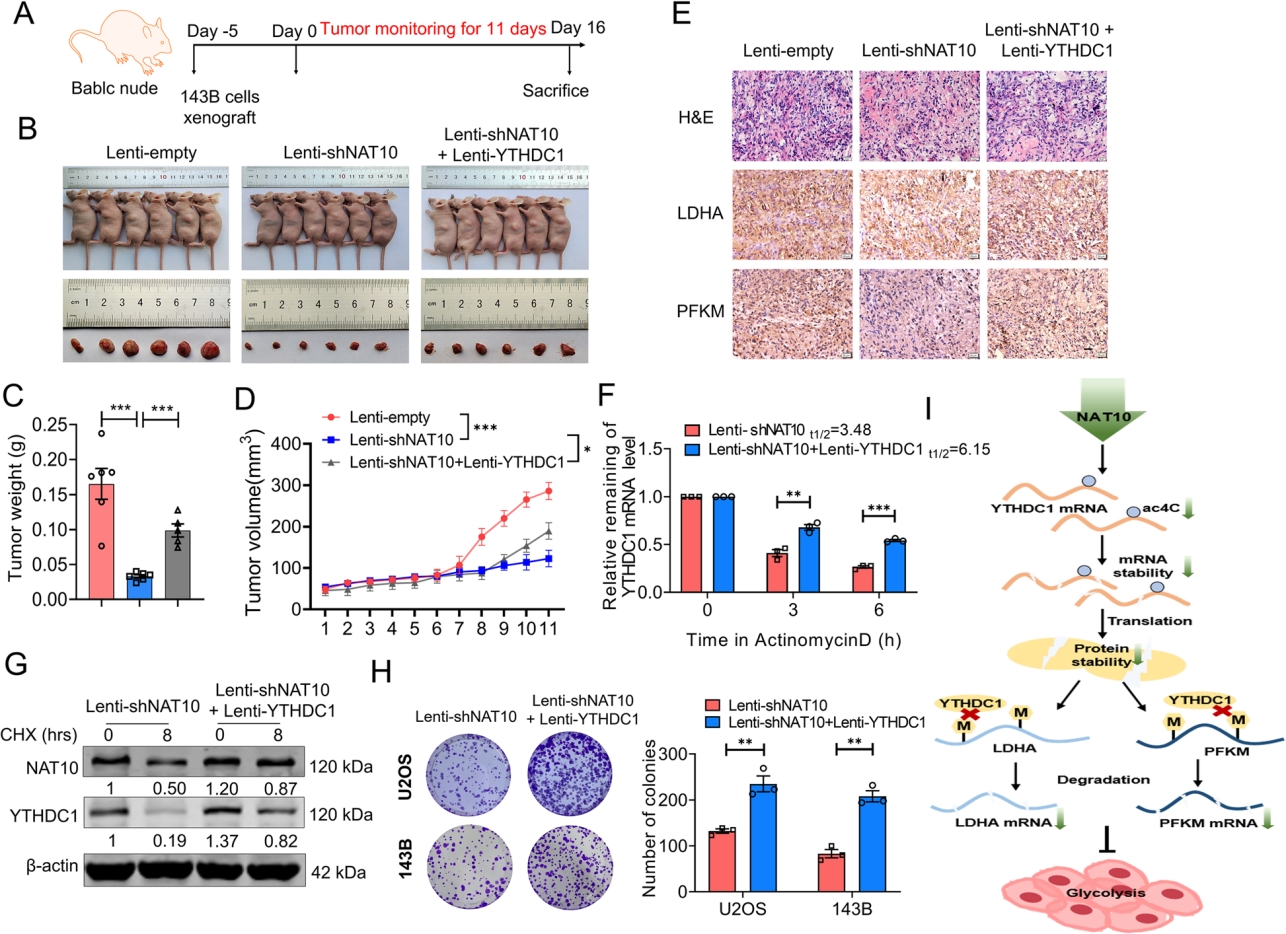
YTHDC1 partially abrogated the inhibitory effect of NAT10 knockdown on tumor growth in vivo. **A** Schematic presentation on the time-line of in vivo cell transplantation. **B** Representative images of tumors with corresponding tumor volumes. **C**, **D** tumor weight and tumor volume in nude mice bearing 143B cells were co-transfected with Lenti-shNAT10/empty constructs and Lenti-YTHDC1. **E** H&E and representative immunohistochemical images of PFKM and LDHA staining from subcutaneous xenograft tissues in 143B tumor-bearing model (Bar: 20 μm). **F** The YTHDC1 mRNA half-life was estimated according to linear regression analysis after the indicated actinomycin D treatment. **G** The 143B cells were transfected with lentiviral vectors containing shNAT10 for 48 h and part of NAT10 knockdown cells were also transfected with YTHDC1 overexpressing lentivirus for 48 h, then treated with cycloheximide (CHX)-mediated for 8 h. Western blot analysis of YTHDC1 protein levels over time in response to DNA damage. **H** Representative images and quantitative results of cancer cell colonies. **I** Schematic illustration on the signaling pathway of NAT10 in glycolysis via targeting signaling axis for YTHDC1-LDHA/PFKM signaling axis in an m^6^A methylation modification manner in osteosarcoma. Data are expressed as mean ± SEM. *P < 0.05; **P < 0.01; ***P < 0.001

To obtain more conclusive evidence that the effects of NAT10 and YTHDC1 on tumor growth, stable NAT10 knockdown and control cells were constructed by lentiviral transfection of 143B cells. A subset of NAT10 knockdown cells were also transfected with YTHDC1 overexpressing lentivirus and used to establish an animal xenograft model (Fig. 6A, B). As depicted in Supplementary Fig. 2A and B, the lentivirus infection efficiency of shNAT10 and YTHDC1 was confirmed through western blotting analysis. We accurately observed the time of tumor formation, and the tumor weight and volume. Our results showed that tumor volumes and weights of cancer tissue were significantly decreased in mice inoculated with lentiviral stable NAT10 knockdown cells as compared with those derived from empty lentivirus transfected, supporting the previous findings (Fig. 6C, D). The tumor volumes of mice with stably transfected NAT10 knockout cells were reduced by 57.1% and the tumor tissue weight was only 0.20 times that of the empty lentivirus transfected. Furthermore, YTHDC1 overexpression treatments significantly restored in vivo tumor growth compared with the Lenti-shNAT10 group. Similarly, these treatment effects were further supported by hematoxylin and eosin (H&E) staining from the differently treated groups (Fig. 6E). Interestingly, IHC staining of glycolytic marker proteins PFKM and LDHA levels showed that NAT10 knockdown significantly inhibited PFKM and LDHA production, whereas overexpression of YTHDC1 significantly reversed this effect (Fig. 6E). These findings indicate that YTHDC1 mediated m^6^A methylation could partially eliminate the inhibitory effect of NAT10 knockdown on tumor growth in vivo. To further explore the potential mechanism of NAT10 regulating YTHDC1 transcripts expression, we investigate the stability of YTHDC1 and cell proliferation by lentiviral overexpressing YTHDC1 under the condition of lentiviral depleting NAT10 at cellular level. We found that Lentiviral overexpression of YTHDC1 partially abrogated the reduced stability of YTHDC1 caused by lentiviral depleting NAT10 in the presence of transcription inhibitor actinomycin D (Act D) (Fig. 6F, G). We found lentiviral overexpression of YTHDC1 partially abrogated the reduced stability of YTHDC1 mRNA and protein caused by lenti-shNAT10 knockdown (Fig. 6F, G). And lentiviral overexpression of YTHDC1 increased in the half-life of YTHDC1 transcripts (from 3.48 to 6.15 h) as compared with the Lenti-shNAT10 group. Meanwhile, lenti-shNAT10 accelerated the degradation of YTHDC1 in the presence of translation inhibitor cycloheximide (CHX), lentiviral overexpression of YTHDC1 partially restored the inhibitory effect of lenti-shNAT10 knockdown. Furthermore, lentiviral depleting NAT10 remarkably inhibited the colony-formation capacity in both U2OS and 143B osteosarcoma cells in colony-formation assays, lentiviral overexpression of YTHDC1 significantly abolished this effect (Fig. 6H).

## Discussion

The N-acetyltransferase 10 (NAT10) enzyme plays a critical role in mediating the modification of N4-acetylcytidine (ac4C) mRNA, which is essential for mRNA stability and translational efficiency. NAT10 has been identified as the only enzyme known to catalyze ac4C modification and is involved in various cellular processes while being associated with a range of diseases [22–25]. Despite its crucial biological function, NAT10 has also been reported to promote tumor development in various cancers. In this study, we demonstrate for the first time that ac4c driven RNA m^6^A modification can positively regulate the glycolysis of cancer cells and reveals a previously unrecognized signaling axis of NAT10/ac4C-YTHDC1/m^6^A-LDHA/PFKM in osteosarcoma. Additionally, our study is the first to reveal that NAT10-mediated YTHDC1 mRNA decay is partially due to the reduced stability of its mRNA transcript, leading to the inhibition of protein translation and suppressing osteosarcoma cell growth. Therefore, our findings suggest that NAT10 serves as an effective and novel therapeutic target for osteosarcoma through YTHDC1-mediated m^6^A modification, which can impact the translation efficiency of LDHA and PFKM. However, less is known about the effects of YTHDC1 on NAT10 levels, and thereby affects oncogenic progression, which we will explore it even further.

Growing evidence suggests that NAT10 dysfunction is associated with several diseases. For instance, NAT10 promotes colon cancer development by regulating mRNA stability and FSP1 expression, thus becoming a new prognostic and therapeutic target [18, 23]. The down-regulation of NAT10 results in the reduction of ac4C modification in specific regions of mRNA, impairing translational efficiency of BCL9L, SOX4, AKT1 as well as the stability of BCL9R and SOX5, thereby reducing bladder cancer burden [24]. In pancreatic ductal adenocarcinoma, the LINC00623\/NAT10 signaling axis promotes cell proliferation, tumorigenicity, migration and invasion. Mechanistically, NAT10 enhances oncogenic mRNA stability and translation in pancreatic ductal adenocarcinoma by remodeling ac4C modified mRNAs [22]. In multiple myeloma cells, NAT10 acetylates and stabilizes BCL-XL mRNA, which leads to elevated expression of BCL-XL, inhibiting apoptosis and activating the PI3K-AKT pathway, thereby promoting cell cycle progression and proliferation during multiple myeloma malignancy [26]. Furthermore, NAT10 directly controls the expression of RUNX2 through ac4C modification, regulating osteoblast differentiation ability of BMSC in vitro; thus, it can be used as a new potential therapeutic target for osteoporosis [27]. Like other diseases, our study reveals that NAT10 is highly expressed in osteosarcoma patients, and its abnormal levels predict disease progression and lower overall survival. Importantly, NAT10 is sufficient and necessary for the tumorigenic properties of osteosarcoma. Therefore, we propose targeting NAT10 as a promising therapeutic strategy for osteosarcoma treatment.

The YTHDC1 transcript has been identified as a critical target gene for ac4C acetylation, which was validated by RIP experiments and subsequent functional studies. The most abundant internal modification of eukaryotic messenger RNA (mRNA) is N6-methyladenosine (m^6^A), which plays an essential role in RNA biology. The m^6^A-selective "reader" protein of the YTH family mediates m^6^A-modified mRNA into RNA metabolic pathways [28]. YTHDC1 belongs to the nuclear m^6^A reader, which regulates mRNA splicing by recruiting and regulating pre-mRNA splicing factors, allowing them to enter the binding region of the targeted mRNA [29, 30]. Additionally, YTHDC1 binds to m^6^A-modified RNA and promotes splice site selection, playing a vital role in promoting the export of methylated mRNA from the nucleus to the cytoplasm in HeLa cells [31]. Studies have shown that YTHDC1 can regulate biological behaviors associated with aerobic glycolysis. For instance, it forms a robust YTHDC1-miR-30d-RUNX1 SLC2A1/HK1 axis to inhibit PDAC initiation and progression [32]. However, in our study, we found that YTHDC1-mediated regulation of nuclear RNA is critical for osteosarcoma development.

Despite significant achievements in the clinical treatment of osteosarcoma in recent years, the overall survival rate of patients has not improved significantly. The metabolic pathways of tumor cells mainly include glycolysis, fat metabolism, glutaminolysis and oxidative phosphorylation [33]. Cancer cells preferentially use glycolysis rather than oxidative phosphorylation to meet their increased energy and biosynthetic demands, known as the Warburg effect. Glycolysis is a driver of multiple cancers and has emerged as a new cancer-targeted therapy [34]. Glycolysis in osteosarcoma is closely related to a variety of oncogenes and tumor suppressor genes, and many signaling pathways have been reported to be involved in the regulation of glycolysis [35, 36]. The increased glycolysis is mainly caused by increased expression of enzymes and transporters involved in glucose uptake, lactate production and secretion. Our article shows that YTHDC1 transcript was a critical target gene for ac4C acetylation, while indeed affects glycolysis in osteosarcoma cells by examining differences in glucose, lactate, and pyruvate content in cells or media after NAT10 knockdown. Moreover, our study shows that NAT10 functions as a cancer-promoting molecule by targeting the glycolysis genes PFKM and LDHA directly or indirectly through acetylation in mRNA to reduce glycolysis breakdown and inhibit tumorigenesis. We observed mRNA for PFKM and LDHA genes carrying multiple potential m^6^A modification sites using the sequence-based SRAMP m^6^A modification site predictor (http://www.cuilab.cn/sramp). Subsequently, we confirmed that PFKM and LDHA could specifically bind YTHDC1 by RIP assay and subsequent experiments, while YTHDC1 could regulate the expression of PFKM and LDHA. Overall, our study sheds new light on the regulation of glycolysis in osteosarcoma by YTHDC1 and provides a promising therapeutic target for this disease.

Molecular therapy targeting NAT10 and YTHDC1 has shown potential for the treatment of various diseases, including osteosarcoma. However, there are several limitations and challenges that need to be addressed before such therapies can be developed effectively and safely. One potential limitation associated with targeting NAT10 is its involvement in various cellular processes, making it difficult to target specifically without affecting healthy cells [37]. Moreover, the exact manner in which NAT10-mediated ac4C promotes target gene stability and translation efficiency is unknown, which adds to the challenge of developing effective and specific inhibitors. Therefore, there is a need to gain a better understanding of NAT10's biological functions to determine the full spectrum of its therapeutic potential. Another challenge that needs addressing is the specificity of YTHDC1 inhibitors needs to be evaluated extensively to avoid off-target effects, given that YTHDC1 is not the only RNA-binding protein involved in alternative splicing and other cellular processes [38]. Furthermore, there are several challenges associated with developing molecular therapies based on NAT10 and YTHDC1. For instance, identifying selective and specific inhibitors and determining their efficacy in preclinical and clinical studies will require significant research efforts. Moreover, the development of effective delivery methods to transport these therapies to targeted cells is still a significant obstacle that needs to be addressed. Despite these challenges, targeting NAT10 and YTHDC1 remains a promising therapeutic strategy for various diseases, including osteosarcoma.

In conclusion, our findings bridge ac4C acetylation modifications with m^6^A methylation modifications, thereby providing new insights into epigenetic regulation in human cancer. Our study demonstrates the critical role of ac4c driven RNA m^6^A modification in the development of osteosarcoma characterized by the induction of glycolytic processes in osteosarcoma cells. We reveal previously unrecognized signaling axis for NAT10/ac4C-YTHDC1/m^6^A-LDHA/PFKM in osteosarcoma, suggesting that reprogramming RNA m^6^A methylation as a potential and promising strategy for osteosarcoma treatment, and providing profound insights into the molecular mechanisms underlying osteosarcoma development. Furthermore, given the functional importance of NAT10 in multiple myeloma as well as osteoporosis, targeting NAT10 signaling through selective promoters may be a promising therapeutic strategy for the treatment of osteosarcoma, particularly in patients with high NAT10 levels.

## Materials and methods

### Cell culture

Human osteosarcoma cancer cell lines 143B and U2OS were cultured in MEM medium supplemented with 10% fetal bovine serum (FBS) (S711-001S Biological Industries, Israel). All cell lines were maintained in an incubator at 37℃ in an atmosphere containing 5% CO_2_.

### Xenograft tumorigenesis model

BABL/c nude mice were purchased from Beijing Vital River Laboratory Animal Technology Limited Company (Beijing, China). The stable NAT10 knockdown and control cells were constructed by lentiviral transfection of 143B cells. A subset of NAT10 knockdown cells were also transfected with YTHDC1 overexpressing lentivirus and used to establish an animal xenograft model. When the cell density reached more than 90%, the cells were digested and centrifuged, mice were injected subcutaneously with 1 × 10^6^ 143B cells to form subcutaneous xenografts. Tumor growth was monitored, and tumor size was measured and calculated according to the standard formula: Volume = (length × width^2^)/2.

### Ac4C dot blot

Total RNA was heated to 75℃ for 5 min, placed on ice for 1 min, and loaded onto Hybond-N + membranes. Membranes were crosslinked with 150 mJ/cm2 in a UV 254 nm Stratalinker 2400 (Stratagene). Then membranes were blocked with 5% nonfat milk in 0.1% Tween 20 PBS (PBST) for 1 h at room temperature and incubated with an anti-ac4C antibody (1:1,000, Abcam, ab253039, Cambridge, UK) in PBST at 4℃ overnight. The membranes were then washed three times with 0.1% PBST, probed with a HRP-conjugated secondary anti-rabbit IgG in PBST (1:1,000) at room temperature for 1 h, and washed three times with 0.1% PBST. Odyssey CLx and quantified with LI-COR Image Studio Software (LI-COR Biosciences, Lincoln, NE, USA) was used to visualize the dots.

### m^6^A enzyme-linked immunosorbent assay (ELISA)

The m^6^A RNA methylation assay kit (ab185912, Abcam, Cambridge, UK) was used to measure the m^6^A content in total RNAs following the manufacture’s protocol. Briefly, 400 ng RNAs were coated on assay wells, and then incubated at 37℃ for 90 min. 50 μL capture antibody solution and 50 μL detection antibody solution was then added to assay wells separately incubated for 60 and 30 min at room temperature. The m^6^A levels were quantified using a microplate reader at a wavelength of 450 nm.

### Cell transfection

Lipofectamine TM 3000 transfection reagent (6 μL) (L3000-015; Invitrogen, USA) and siRNA (20 nM) were respectively diluted in 125 μL Opti-MEM medium and incubated for 2 min (31985–070, Gibco, USA). The two diluted regents were added to the cells after incubation for 10 min. Subsequent experiments were carried out 24 h after transfection. The siNAT10 and siYTHDC1 were obtained from GenePharma (China).

The sequences of the constructs used in this study were

- NAT10 siRNA sense: 5’-CAGCACCACUGCUGAGAAUAAGA-3’ and
- NAT10 siRNA antisense: 5’-UCUUAUUCUCAGCAGUGGUGCUG-3’;
- YTHDC1 siRNA sense: 5’-GCAAGGAGUGUUAUCUUAATT-3’ and
- YTHDC1 siRNA antisense: 5’-UUAAGAUAACACUCCUUGCTT-3’;
- siNC sense: 5’-ACGUGACACGUUCGGAGAATT-3’ and
- siNC antisense: 5’-UUAAGAUAACACUCCUUGCTT-3’.

The lentivirus vectors with shNAT10, YTHDC1 and negative control (NC) were constructed by HANbio biotechnology (China). Lentivirus infection were performed according to standard protocols as recommended by the manufacturer. 5 × 10^5^ 143B cells were inoculated in the cell vial, lentiviral infection could be performed when the density reached 40%-50%. The volume of lentivirus needed was calculated according to the virus titer and MOI (The volume of lentivirus = MOI x Cell number/Virus titer × 10 00, Lenti-shNAT10 MOI = 40, Virus titer = 3 × 10^8^ TU/ml; Lenti-YTHDC1 MOI = 14.4, Virus titer = 1 × 10^8^ TU/ml). Polybrene was added to each cell vial at the rate of 10 μg/ml to help enhance the infection efficiency, after 6 h, 4 ml pure culture medium was added for culture. After 24 h, the culture was continued with complete culture medium. A portion of shNAT10 infected cells were reinfected with YTHDC1 overexpressing lentivirus, as above.

### RNA stability assay

143B cells were cultured in 6 well plates and transfected with desired constructs as described above. After 24 h transfection, cells were treated with actinomycin D (Act D, 10 μg/mL, GC16866, GLPBIO, USA) for 0 h, 3 h and 6 h before collection. Total RNAs were isolated for qRT-PCR analysis.

### Protein stability assay

143B cells were plated at a density of 2 × 10^6^ cells and transfected for 24 h. Cells were treated with actinomycin D (Act D, 10 μg/mL, Cat# GC16866, GLPBIO, USA) for 0 and 8 h. Protein levels of YTHDC1 were analyzed by Western blotting.

### Ac4C RNA immunoprecipitation (RIP)-qPCR

RIP assay was carried out in 143B cells using Magna RIP Kit (17–700, Millipore, USA) following the manufacturer instructions. In brief, a sufficient number of 143B cells (more than 2 × 10^7^ cells per sample) were lysed by RIP lysis buffer, magnetic beads pre-coated with 5 μg ac4C antibody or mouse IgG (Millipore) was incubated with sufficient cell lysates at 4℃ overnight. The mixture was digested with proteinase K before the immunoprecipitated RNAs were extracted, purified and subjected to qPCR.

### Western blot

Total protein was extracted from the cells using 1 × RIPA lysis buffer (P10013B, Beyotime, Shanghai, China). The concentration of protein was measured by using BCA Protein Assay Kit (P0010, Beyotime, Shanghai, China). And then equal amounts of proteins were separated by SDS-PAGE and transferred to nitrocellulose filter membrane (NC membrane). After electroblotting, the membranes were blocked with PBS-5% fat-free dried milk at room temperature for 1 h and incubated with primary antibodies for LDHA (1:2000, Santa Cruz sc-137243, Texas, USA), PFKM (1:1000, ABclonal A5477, Shanghai, China), NAT10 (1:1000, ABclonal A19286, Wuhan, China), YTHDC1 (1:500, Proteintech 14,392–1-AP, Wuhan, China), ac4C (1:1000, Abcam ab253039, Cambridge, UK) or β-actin (1:5000, Abcam ab253039, Cambridge, UK). The membrane was then incubated with anti-mouse secondary antibodies (RS23910, ImmunoWay, USA) or anti-rabbit secondary antibodies (RS23920, ImmunoWay, USA). Results were detected using Odessey Clex (LI-COR, USA), followed by further analysis.

### Quantitative real-time polymerase chain reaction (qRT-PCR)

Trizol reagent (GK20008, GLPBIO, USA) was used to extract total RNAs. High-Capacity cDNA Reverse Transcription Kit (Cat#43744967; Thermo Fisher Scientific, Waltham, USA) was used to synthesize cDNA. qRT-PCR analysis was performed using 1 μL cDNA, 1 μL forward primer, 1 μL reverse primer, and FastStart Universal SYBR Green PCR Master (Cat#04913914001; Roche, Switzerland) by a 7500 Fast Real-Time instrument (Roche, Switzerland). The expression levels of the target genes were normalized to GAPDH gene. The primer pair sequences used in the present study are listed in Supplementary Table 1.

### Ethynyl-2-deoxyuridine (EdU) staining assay

Cell proliferation was determined by a EdU Apollo DNA in vitro kit (C10310-1 Ribobio, China). Briefly, the cells (2 × 10^5^/mL seeded in a 24-well plate) were treated with 30 μM/mL EdU at 37℃ for 90 min, after fixing with 4% paraformaldehyde (m/v) for 30 min. And permeabilization in 0.5% Triton X-100, the Apollo staining solution was added to the plate in dark for 30 min. Then, the cells were incubated with 4′,6-diamidino-2-phenylindole (DAPI, 20 μg/mL) for 10 min. The average ratio of EdU positive cells to total cells was calculated in randomly selected areas under a microscope (Olympus).

### Colony formation assay

Transfected cells were seeded into 6-well plates (1500 cells/well) and kept in MEM containing 10% FBS for 14 days, at 37℃. Colonies were stained with 0.1% crystal violet for 20 min after fixation with 4% formaldehyde for 30 min. The visible colonies were counted.

### Invasion assay

Matrigel invasion assay was performed using 24-well plates inserted by Matrigel (BD Biosciences, USA) pre-coated 24 mm Transwell® chambers (Corning, USA). 1 × 10^5^ cells were suspended in 200 μL serum-free MEM Medium and added to the upper chamber. The bottom of the well was refilled with MEM Medium containing 10% FBS. After 24 h, cells on the lower side of the membrane were immobilized and stained with 0.1% crystal violet (C0121, Beyotime, China) for 20 min and counted under a light microscopy (ECLIPSE TS100, Nikon).

### Migration assay

Scratch experiments were performed to detect the migration of U2OS and 143B cells. Cells were plated into 6 well plates at a density of 2.5 × 10^5^ cells/mL and incubated for 24 h. When the confluence of cells reached 70%, a 200 μL sterile pipette tip was used to make vertical scratches in each hole. Cells were washed with PBS and then transfected with siRNAs. Images were taken with standard light microscopy (ECLIPSE TS100, Nikon, Japan) to observe the degree of migration at 0 h, 24 h, and 48 h.

### Cell Counting Kit-8 (CCK8) assay

Cell viability was detected by the Cell Counting Kit-8. Briefly, transfected U2OS and 143B cells were seeded into 96-well plates overnight at 37℃. After treatment for 24 h, 10 μL of CCK-8 solution was added to each well and then cultured for 90 min at 37℃. The absorbance was measured using a microplate reader (Infinite 200 Pro, TECAN) at 450 nm.

### ATP production assay

ATP Assay Kit (Ab83355, Abcam, Cambridgeshire, UK) was used to measure cellular ATP contents according to the manufacturer’s protocol. U2OS and 143B cells were seeded into 24-well plate. Firstly, ATP standards of 50 µL were prepared to obtain standard curve. Briefly, centrifuged samples, collected supernatants and transferred to new tubes. 100 μL of the cell lysate was mixed with 100 μL of ATP reaction mix and incubated for 30 min. Absorbance was measured using a microplate reader (Infinite 200 Pro, TECAN) at 570 nm wavelength.

### Glucose uptake assay

Glucose Uptake Colorimetric Assay Kit (Ab136955, Abcam, Cambridgeshire, UK) was used to determine glucose uptake according to the manufacturer’s protocols. 5 × 10^6^ cells were plated into 6-well cell culture plates and incubated at 37℃ overnight. After transfection for 24 h, complete medium was replaced with serum-free medium for starvation treatment. After 4 h, the cells were digested and centrifuged, and 200 μL of supernatant was taken and incubated with the detection solution for 30 min. At this time, glucose was oxidized to gluconic acid and hydrogen peroxide by glucose oxidase. In the presence of peroxidase, hydrogen peroxide will react with o-dianisidine to form a color product. When sulfuric acid is added, the oxidized o-dianisidine continues to react with sulfuric acid to form more stable color products. The pink signal intensity was measured using a microplate reader (Infinite 200 Pro, TECAN) at the wavelength of 540 nm, which is directly proportional to the original glucose concentration in the sample.

### Pyruvate uptake assay

Micro Pyruvate Acid (PA) Assay Kit (KTB1121, Abbkine, USA) was used for the determination of Pyruvate in cells. 24 h after the transfection of 143B and U2OS cells, collect 5 × 10^6^ cells into a centrifuge tube, discard the supernatant, add 1 mL Extraction Buffer, ultrasonic crush for 5 min (ultrasonic wave for 3 s, with interval of 7 s, repeat for 30 times), allow to stand for 30 min, centrifuge at room temperature at 4000 × g for 10 min, take the supernatant, and place on ice for testing. The absorbance was measured at 520 nm to show difference in pyruvate content.

### Lactate production assay

Lactate Colorimetric Assay Kit (colorimetric) for the determination of lactic acid production (ab65331, Abcam, UK). 24 h after transfection of 143B and U2OS cells, the culture medium was replaced with serum-free medium. After 4 h of starvation, the supernatant was collected and the content of lactic acid was determined. The D-lactate concentration assay was performed following the manufacturer’s instruction, added 50 μL solution to each well, incubated dark at room temperature for 30 min, and determined lactate level with a microplate reader (Infinite 200 Pro, TECAN) at 450 nm.

### Hematoxylin and Eosin (H&E) staining

The slides were baked at 60℃ for 2 h, then followed by dewaxed and washed with PBS. The H&E staining Kit (G1120, Solarbio, China) was used for H&E staining, paraffin sections were stained with hematoxylin for 7 min and differentiated with differentiation solution for 10 s. And then the sections were stained with eosin, followed by dehydrated with gradient alcohol and cleared with xylene. Images were acquired under a microscope (Olympus).

### Immunohistochemistry (IHC)

The processed tissues were embedded in paraffin, cut into 4 μm sections using a Leica RM2255 (Leica Biosystems, UK). The slides were baked at 60℃ for 2 h, then followed by dewaxed and washed with PBS. The tissues were blocked with 3% H_2_O_2_ at RT for 10 min. Antigen retrieval was carried out in sodium citrate buffer or EDTA for 20 min at boiling temperature. Then added different primary antibodies LDHA (1:2000, Santa Cruz sc-137243, Texas, USA), PFKM (1:1000, ABclonal A5477, Shanghai, China) at 4℃ overnight. Next day, incubated with horseradish peroxidase-conjugated goat anti-rabbit antibody (GB23303, Servicebio, China), DAB working solution was incubated for 1.5 min and stopped by distilled water washing. Then counterstained with hematoxylin. The sections were dried at 37℃ overnight and the images were captured under a fluorescent microscope (Olympus).

### Statistical analysis

All experimental results were repeated at least three times and were expressed as means ± SEM. Statistical analyses were performed using GraphPad Prism 8.0. Two-tailed Student' s t-test was used for two-group comparisons and one-way analysis of variance (ANOVA) followed by Tukey’s post-hoc correction for multigroup comparisons. P < 0.05 was considered statistically significant: *P < 0.05; **P < 0.01; ***P < 0.001.

### Supplementary Information

**Additional file 1:** **Supplementary Figure 1.** Immunohistochemistry (IHC) analysis of NAT10 protein expression in normal bone tissues (*n*=20) and osteosarcoma (*n*=71) tissue microarrays (TMAs). **Supplementary Figure 2.** **A** The 143B cells were transfected with lentiviral vectors containing either empty or shNAT10 for 48 h. The protein levels of NAT10 and YTHDC1 were evaluated by western blotting. **B** The 143B cells were transfected with lentiviral vectors containing either empty or YTHDC1 for 48 h. The overexpression efficiency of YTHDC1 was evaluated by western blotting. **Supplementary Table 1.** Primer sequences for qRT-PCR.

**Additional file 2:**

## Data Availability

Not applicable.

## References

[1] Gill J, Gorlick R (2021). Advancing therapy for osteosarcoma. Nat Rev Clin Oncol.

[2] Chen C (2021). Immunotherapy for osteosarcoma: fundamental mechanism, rationale, and recent breakthroughs. Cancer letters.

[3] Corre I (2020). The osteosarcoma microenvironment: a complex but targetable ecosystem. Cells.

[4] Tang BL (2020). Glucose, glycolysis, and neurodegenerative diseases. J Cell Physiol.

[5] Zhang H (2020). Dynamic landscape and evolution of m^6^A methylation in human. Nucleic Acids Res.

[6] Oerum S (2021). A comprehensive review of m^6^A/m^6^A mRNA methyltransferase structures. Nucleic Acids Res.

[7] Tang Y (2021). m6A-Atlas: a comprehensive knowledgebase for unraveling the N6-methyladenosine (m^6^A) epitranscriptome. Nucleic acids research..

[8] Lv D (2022). M6A demethylase FTO-mediated downregulation of DACT1 mRNA stability promotes Wnt signaling to facilitate osteosarcoma progression. Oncogene.

[9] Chen S (2020). WTAP promotes osteosarcoma tumorigenesis by repressing HMBOX1 expression in an m6A-dependent manner. Cell death Dis.

[10] Yang Z (2022). ALKBH5 regulates STAT3 activity to affect the proliferation and tumorigenicity of osteosarcoma via an m6A-YTHDF2-dependent manner. EBioMedicine.

[11] Yuan Y (2021). ALKBH5 suppresses tumor progression via an m6A-dependent epigenetic silencing of pre-miR-181b-1/YAP signaling axis in osteosarcoma. Cell Death Dis.

[12] Arango D (2018). Acetylation of Cytidine in mRNA promotes translation efficiency. Cell.

[13] Thomas JM (2018). A chemical signature for Cytidine acetylation in RNA. J Am Chem Soc.

[14] Bartee D (2022). Site-Specific synthesis of N4-Acetylcytidine in RNA reveals physiological duplex stabilization. J Am Chem Soc..

[15] Liu X (2016). NAT10 regulates p53 activation through acetylating p53 at K120 and ubiquitinating Mdm2. EMBO Reports.

[16] Cui L (2022). RNA modifications: importance in immune cell biology and related diseases. Signal Transduct Target Ther.

[17] Nance KD (2022). Cytidine acetylation yields a hypoinflammatory synthetic messenger RNA. Cell Chem Biol.

[18] Zheng X (2022). N-acetyltransferase 10 promotes colon cancer progression by inhibiting ferroptosis through N4-acetylation and stabilization of ferroptosis suppressor protein 1 (FSP1) mRNA. Cancer Commun (Lond).

[19] Arango D (2018). Acetylation of Cytidine in mRNA promotes translation efficiency. Cell.

[20] Feng Z (2022). The roles of glycolysis in osteosarcoma. Frontiers Pharmacology.

[21] Gao W (2021). The role of S-nitrosylation of PFKM in regulation of glycolysis in ovarian cancer cells. Cell Death Disease.

[22] Feng Z (2022). The LINC00623/NAT10 signaling axis promotes pancreatic cancer progression by remodeling ac4C modification of mRNA. J Hematol Oncol.

[23] Zheng X (2022). N-acetyltransferase 10 promotes colon cancer progression by inhibiting ferroptosis through N4-acetylation and stabilization of ferroptosis suppressor protein 1 (FSP1) mRNA. Cancer Commun (Lond).

[24] Wang G (2022). NAT10-mediated mRNA N4-acetylcytidine modification promotes bladder cancer progression. Clin Transl Med.

[25] Zhang H (2022). NAT10 regulates neutrophil pyroptosis in sepsis via acetylating ULK1 RNA and activating STING pathway. Commun Biol.

[26] Zhang Y (2022). NAT10 acetylates BCL-XL mRNA to promote the proliferation of multiple myeloma cells through PI3K-AKT pathway. Front Oncol.

[27] Yang W (2021). ac4C acetylation of RUNX2 catalyzed by NAT10 spurs osteogenesis of BMSCs and prevents ovariectomy-induced bone loss. Mol Ther Nucleic Acids.

[28] Liu Z (2018). Link between m^6^A modification and cancers. Frontiers Bioengineering Biotechnology.

[29] Roundtree IA, et al. YTHDC1 mediates nuclear export of N6-methyladenosine methylated mRNAs. Elife. 2017;6:e31311. 10.7554/eLife.31311 PMC5648532 28984244

[30] Jiang X (2021). The role of m^6^A modification in the biological functions and diseases. Signal Transduct Target Ther.

[31] Xiao W (2016). Nuclear m(6)A Reader YTHDC1 regulates mRNA splicing. Mol Cell.

[32] Hu Q (2019). UHRF1 promotes aerobic glycolysis and proliferation via suppression of SIRT4 in pancreatic cancer. Cancer Lett.

[33] Tian K (2022). Signature constructed by glycolysis-immune-related genes can predict the prognosis of osteosarcoma patients. Investigational New Drugs.

[34] Ganapathy-Kanniappan S, Geschwind JF (2013). Tumor glycolysis as a target for cancer therapy: progress and prospects. Mol Cancer.

[35] Yu B (2021). The novel prognostic risk factor STC2 can regulate the occurrence and progression of osteosarcoma via the glycolytic pathway. Biochem Biophys Res Commun.

[36] Feng Z, Ou Y, Hao L (2022). The roles of glycolysis in osteosarcoma. Front Pharmacol.

[37] Wang G (2022). NAT10-mediated mRNA N4-acetylcytidine modification promotes bladder cancer progression. Clin Transl Med.

[38] Jiang X (2021). The role of m^6^A modification in the biological functions and diseases. Signal Transduct Target Ther.

