# Supplementary material for: NAT10 mediated ac4C acetylation driven m6A modification via involvement of YTHDC1-LDHA/PFKM regulates glycolysis and promotes osteosarcoma
Source: Cell Commun Signal. 2024 Jan 17;22:51. doi: 10.1186/s12964-023-01321-y (PMC10795323; doi:10.1186/s12964-023-01321-y)
Supplement: Supplementary file 2 — Additional file 1: Supplementary Figure 1. Immunohistochemistry (IHC) analysis of NAT10 protein expression in normal bone tissues (n=20) and osteosarcoma (n=71) tissue microarrays (TMAs). Supplementary Figure 2. A The 143B cells were transfected with lentiviral vectors containing either empty or shNAT10 for 48 h. The protein levels of NAT10 and YTHDC1 were evaluated by western blotting. B The 143B cells were transfected with lentiviral vectors containing either empty or YTHDC1 for 48 h. The overexpression efficiency of YTHDC1 was evaluated by western blotting. Supplementary Table 1. Primer sequences for qRT-PCR. [file 12964_2023_1321_MOESM1_ESM.docx]

Supplementary Data

**NAT10 mediated ac4C acetylation driven m^6^A modification via involvement of YTHDC1-LDHA/PFKM regulates glycolysis and promotes osteosarcoma**

Zhongting Mei^2*^, Zhihua Shen^3,5*^, Jiaying Pu^3^^,5*^, Qian Liu^2^, Guoxin Liu^2^, Xuting He^3,5^, Yang Wang^3,5^, Jinrui Yue^3,5^, Shiyu Ge^2^, Tao Li^2^, Ye Yuan^3,4,5^, Lei Yang^1,6,7^

**Correspondence to:**

Prof. Lei Yang, Department of Orthopedics, The First Affiliated Hospital of Harbin Medical University, Harbin 150001; yangray83@vip.qq.com;

Dr. Ye Yuan, Department of Pharmacy (The University Key Laboratory of Drug Research, Heilongjiang Province), The Second Affiliated Hospital of Harbin Medical University, Harbin, China; yuanye_hmu@126.com;

**
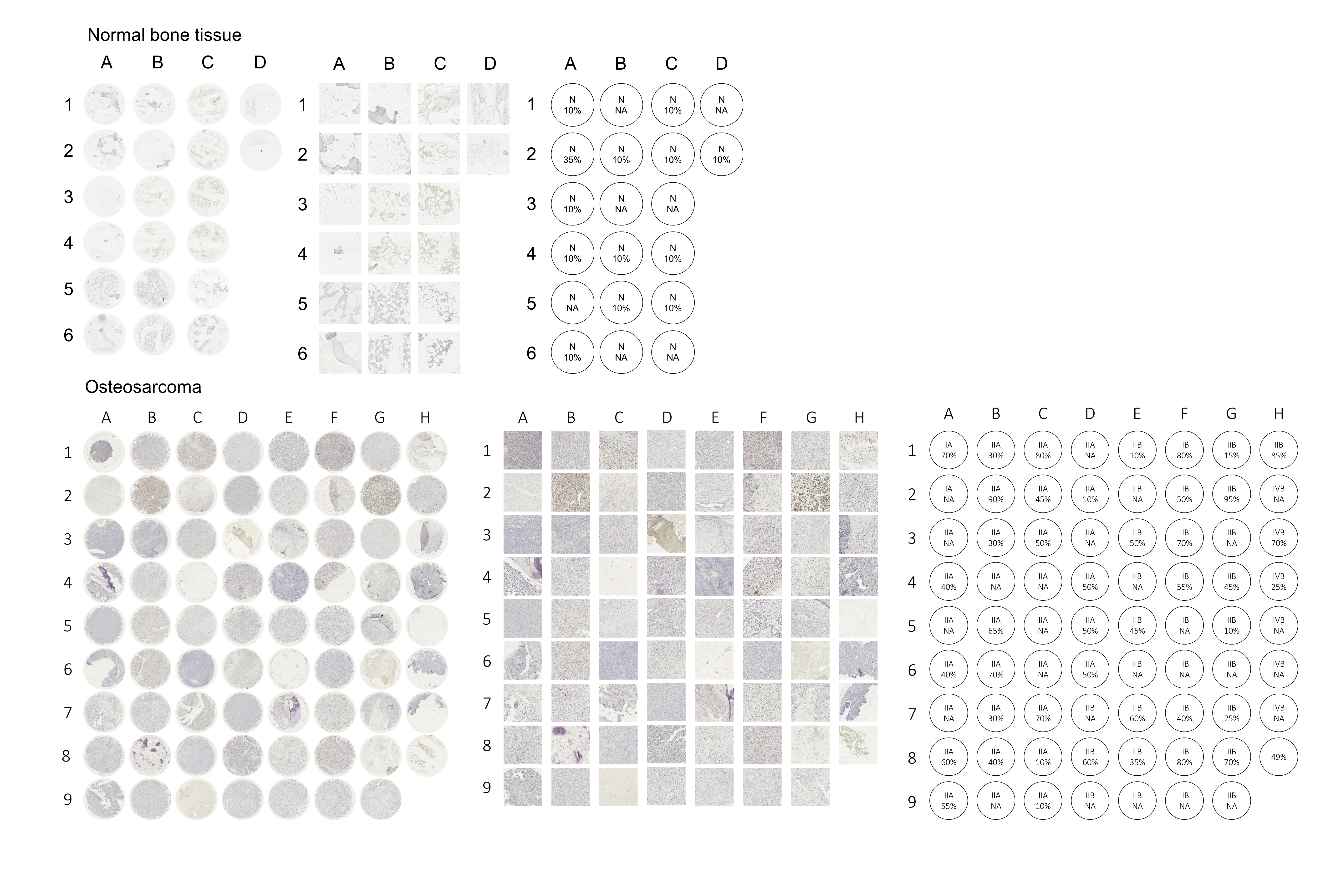
**

**Supplementary Figure 1.**

Immunohistochemistry (IHC) analysis of NAT10 protein expression in normal bone tissues (n=20) and osteosarcoma (n=71) tissue microarrays (TMAs).

B

A


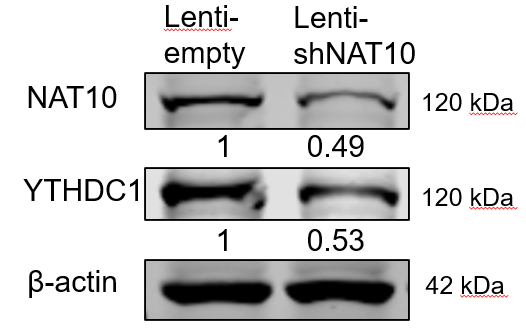

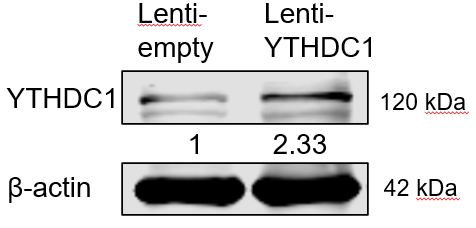


**Supplementary Figure 2.**

**A** The 143B cells were transfected with lentiviral vectors containing either empty or shNAT10 for 48 h. The protein levels of NAT10 and YTHDC1 were evaluated by western blotting. **B** The 143B cells were transfected with lentiviral vectors containing either empty or YTHDC1 for 48 h. The overexpression efficiency of YTHDC1 was evaluated by western blotting.

Supplementary Table 1. Primer sequences for qRT-PCR

| **Primers** | **Primer sequences (5'-3')** |
| --- | --- |
| Human-GLUT1 | F-GAAGTAGGTGAAGATGAAGAACAGAAC  R-CATCCCATGGTTCATCGTGGCTGAACT |
| Human-PFKM | F-ATTGTGCCAGCATCTTCAGCATGAG  R-GGAGAAGCTGCGCGAGGTTTAC |
| Human-GAPDH | F- CAAAGGTGGAGGAGTGGGTGTCGC  R-TTCCGTGTCCCCACTGCCAACGT |
| Human-PGK1 | F-GCGGAGGTTCTCCAGCA  R-ATGTCGCTTTCTAACAAGCTGA |
| Human-PGM1 | F-TTCCATGGCTTTGCGCACCGTCT  R-GGAAACGTGTACTGATTGCAGCCC |
| Human-ENO1 | F-GGTCATCGGGAGACTTGAA  R-GACTTGGCTGGCAACTCTG |
| Human-ENO2 | F-ATGTCCGGCAAAGCGAGCTTCATC  R-TCATGGTGAGTCATCGCTCAGGAG |
| Human-PKM2 | F-TGGTGAGGACGATTATGGCCC  R-GCCCGTGAGGCAGAGGCTGC |
| Human-LDHA | F-GCAACTTGCAGTTCGGGC  R-ATGGCAACTCTAAAGGATCA |
| Human-HK2 | F-GTGAGGATGTAGCTTGTAGAGGGTCCC  R-GCCATCCTGCAACACTTAGGGCTTGAG |
| Human-PFKM | F-ATTGTGCCAGCATCTTCAGCATGAG  R-GGAGAAGCTGCGCGAGGTTTAC |
| Human-PGAM1 | F-TTCCATGGCTTTGCGCACCGTCT  R-GGAAACGTGTACTGATTGCAGCCC |
| Human-YTHDC1 | F-AACTGGTTTCTAAGCCACTGAGC  R-GGAGGCACTACTTGATAGACGA |
| 18S | F-CCTGGATACCGCAGCTAGGA  R-GCGGCGCAATACGAATGCCC |
