# Supplementary material for: NAT10 mediated ac4C acetylation driven m6A modification via involvement of YTHDC1-LDHA/PFKM regulates glycolysis and promotes osteosarcoma
Source: Cell Commun Signal. 2024 Jan 17;22:51. doi: 10.1186/s12964-023-01321-y (PMC10795323; doi:10.1186/s12964-023-01321-y)
Supplement: Supplementary file 3 — Additional file 2: [file 12964_2023_1321_MOESM2_ESM.pptx]

## Slide 1
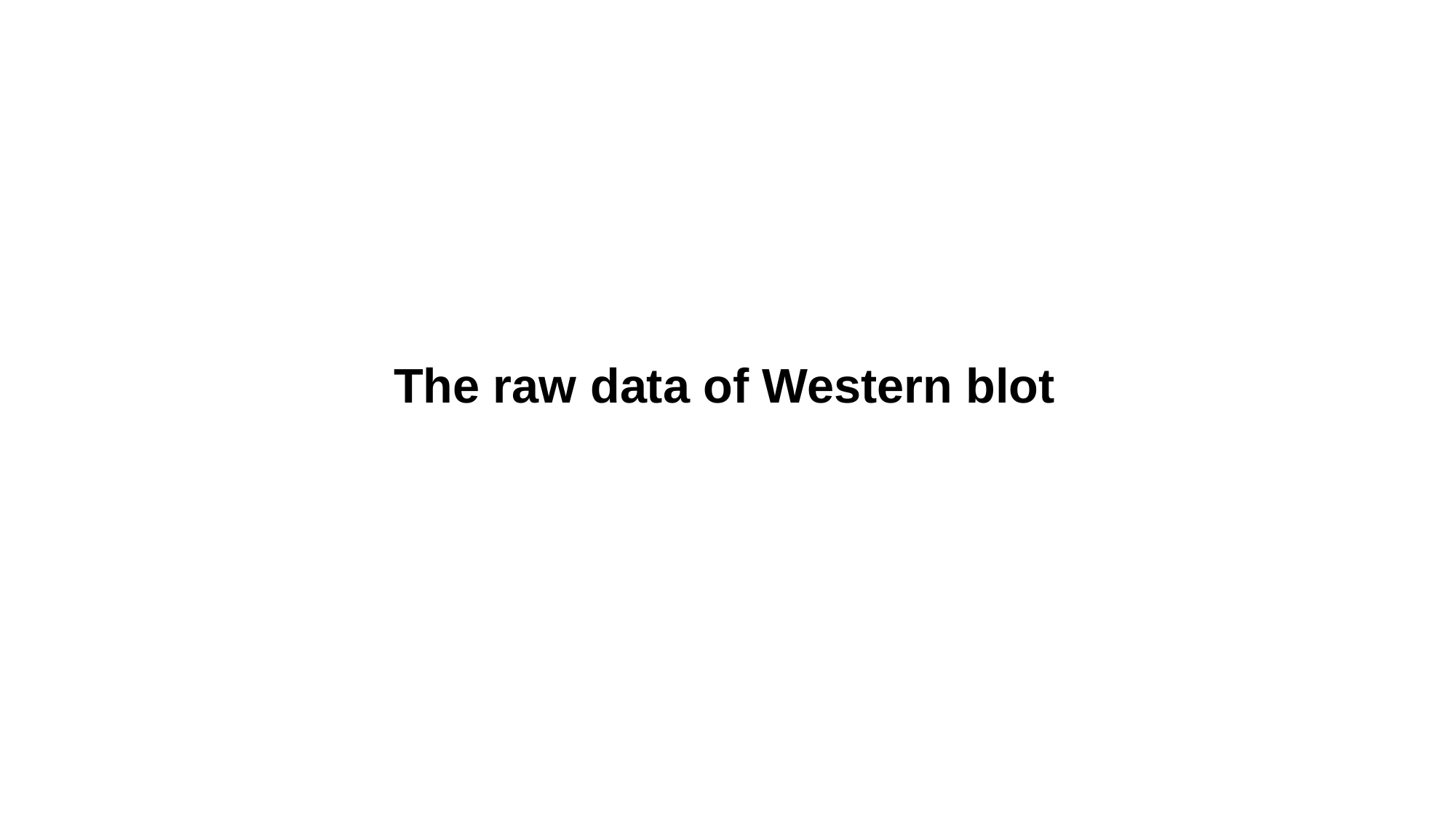

The raw data of Western blot

## Slide 2
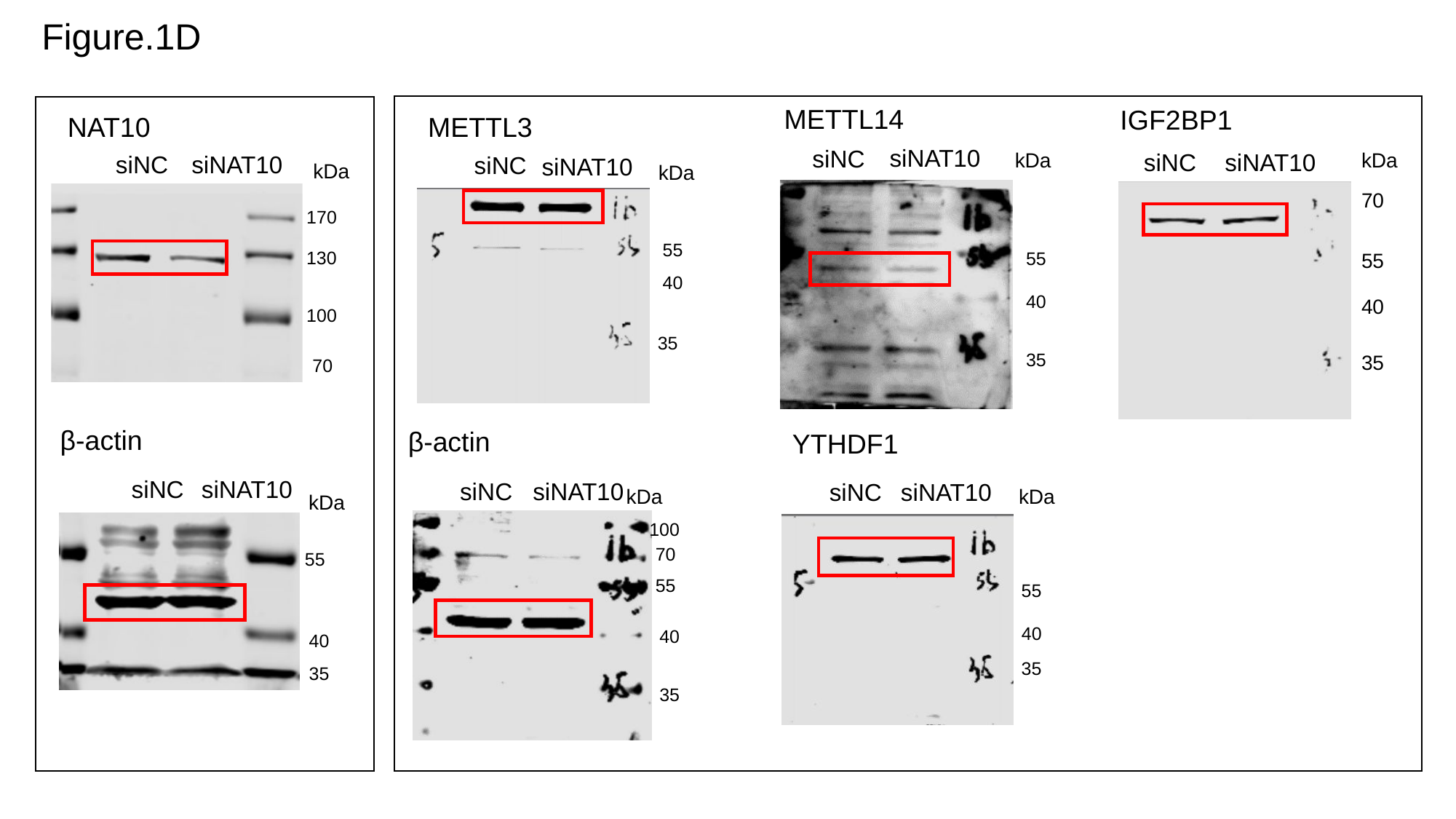

Figure.1D
METTL14
siNAT10
siNC
kDa
55
40
35
IGF2BP1
siNAT10
kDa
siNC
70
55
40
35
NAT10
METTL3
siNC
siNAT10
kDa
55
40
35
siNAT10
siNC
kDa
170
130
100
70
β-actin
siNAT10
siNC
kDa
55
40
35
β-actin
YTHDF1
siNAT10
siNC
55
40
35
kDa
siNAT10
siNC
kDa
100
70
55
40
35

## Slide 3
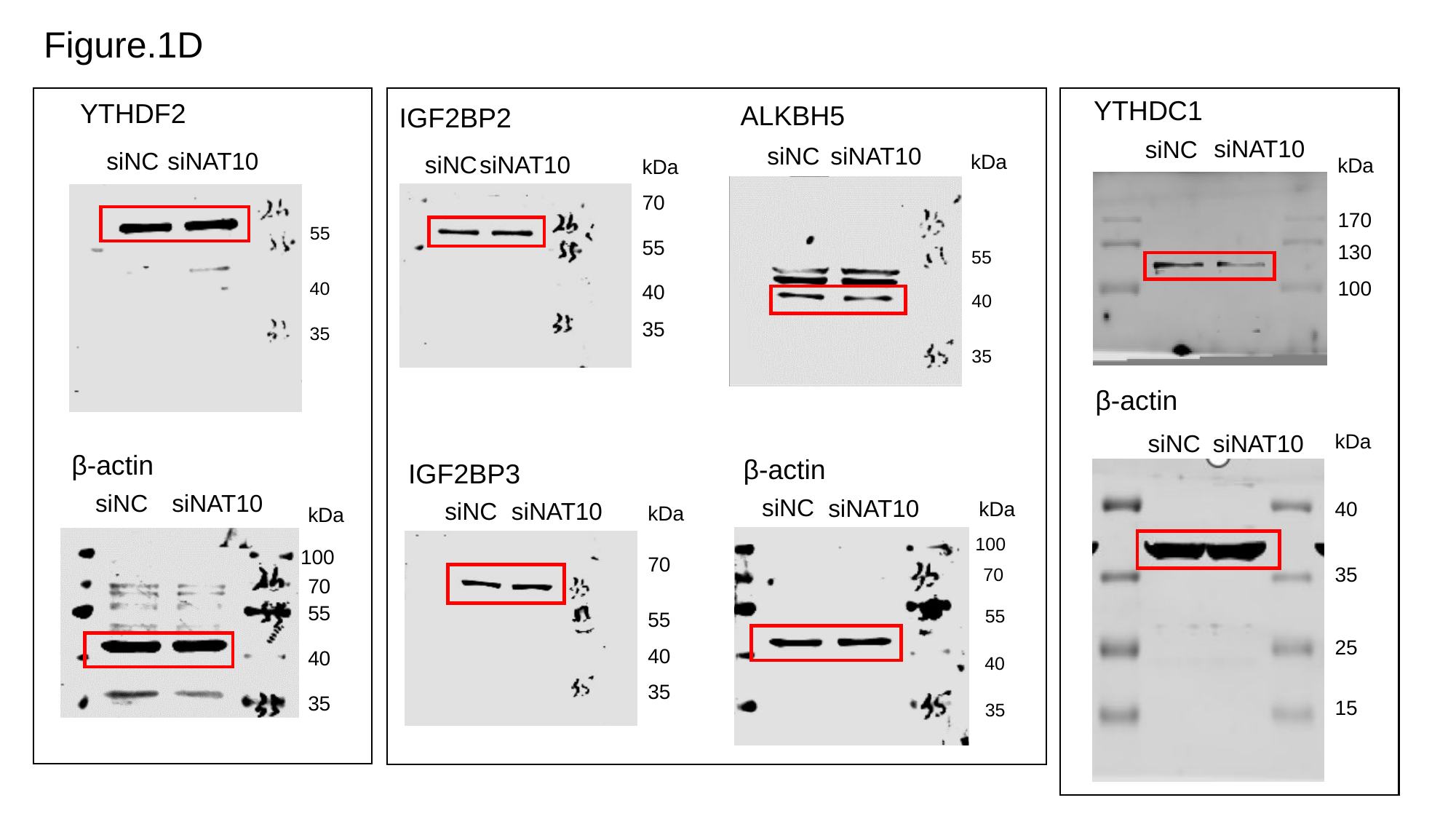

Figure.1D
YTHDC1
siNAT10
siNC
kDa
170
130
100
YTHDF2
siNAT10
siNC
55
40
35
ALKBH5
siNAT10
siNC
kDa
55
40
35
IGF2BP2
siNAT10
siNC
kDa
70
55
40
35
β-actin
siNAT10
kDa
siNC
40
35
25
15
β-actin
siNAT10
siNC
kDa
100
70
55
40
35
β-actin
siNC
siNAT10
kDa
100
70
55
40
35
IGF2BP3
siNAT10
siNC
kDa
70
55
40
35

## Slide 4
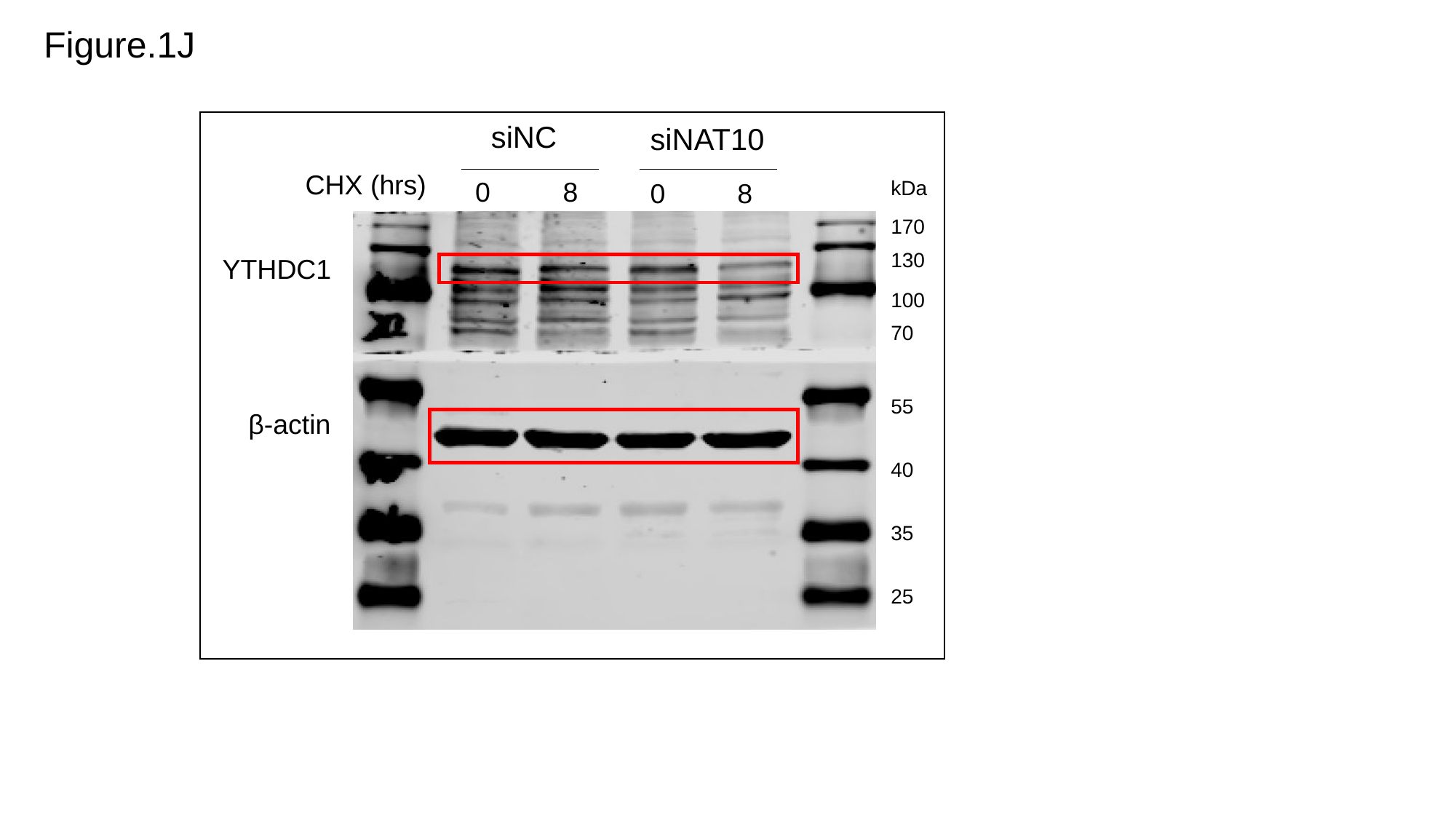

Figure.1J
siNC
siNAT10
CHX (hrs)
kDa
0
8
0
8
170
130
YTHDC1
100
70
55
β-actin
40
35
25

## Slide 5
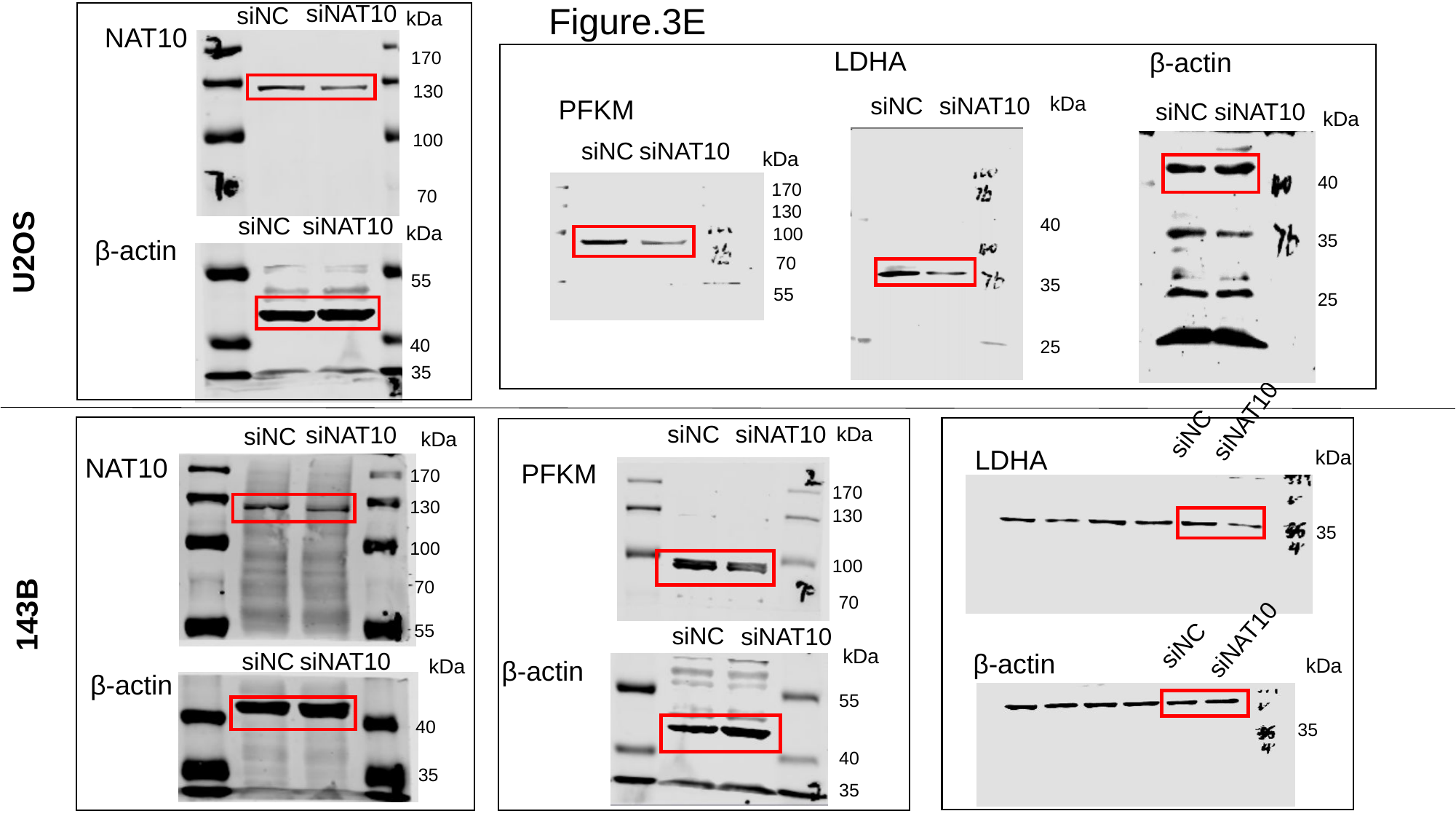

siNAT10
siNC
kDa
170
130
100
70
NAT10
siNAT10
siNC
β-actin
55
40
35
kDa
siNAT10
siNC
kDa
NAT10
170
130
100
70
55
siNAT10
siNC
kDa
β-actin
40
35
Figure.3E
LDHA
siNAT10
kDa
siNC
40
35
25
β-actin
siNAT10
siNC
kDa
40
35
25
PFKM
siNAT10
siNC
170
130
100
70
55
kDa
U2OS
siNAT10
siNC
siNAT10
siNC
kDa
LDHA
kDa
PFKM
170
130
35
100
143B
70
siNAT10
siNC
siNC
siNAT10
kDa
β-actin
kDa
β-actin
55
35
40
35

## Slide 6
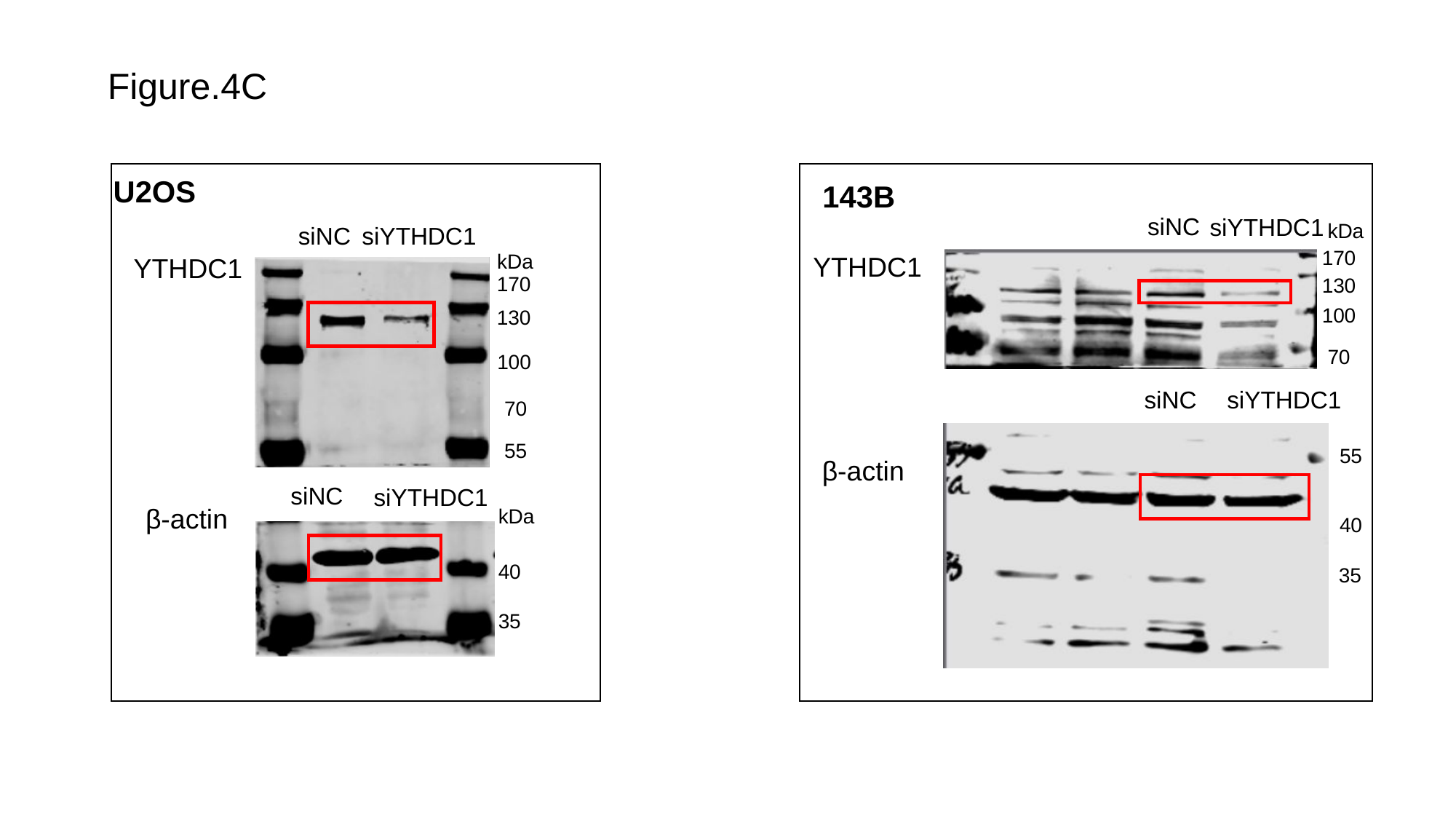

Figure.4C
U2OS
143B
 siNC
 siYTHDC1
kDa
170
YTHDC1
130
100
70
 siNC
 siYTHDC1
55
β-actin
40
35
 siNC
 siYTHDC1
kDa
YTHDC1
170
130
100
70
55
 siNC
 siYTHDC1
β-actin
kDa
40
35

## Slide 7
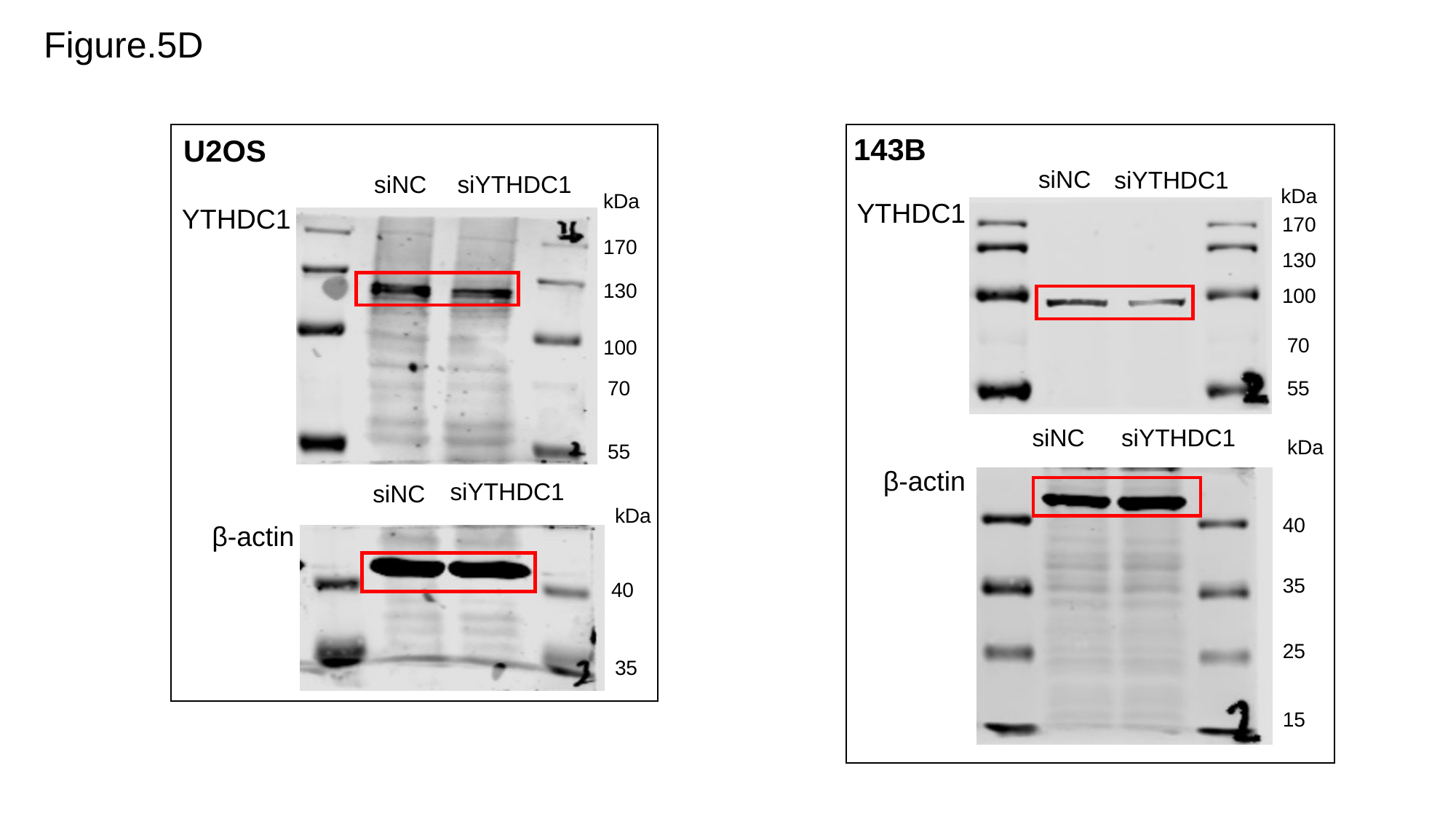

Figure.5D
143B
U2OS
siNC
siYTHDC1
YTHDC1
170
130
100
70
55
siNC
siYTHDC1
kDa
YTHDC1
170
130
100
70
55
kDa
siNC
siYTHDC1
kDa
β-actin
40
35
25
15
siYTHDC1
siNC
kDa
β-actin
40
35

## Slide 8
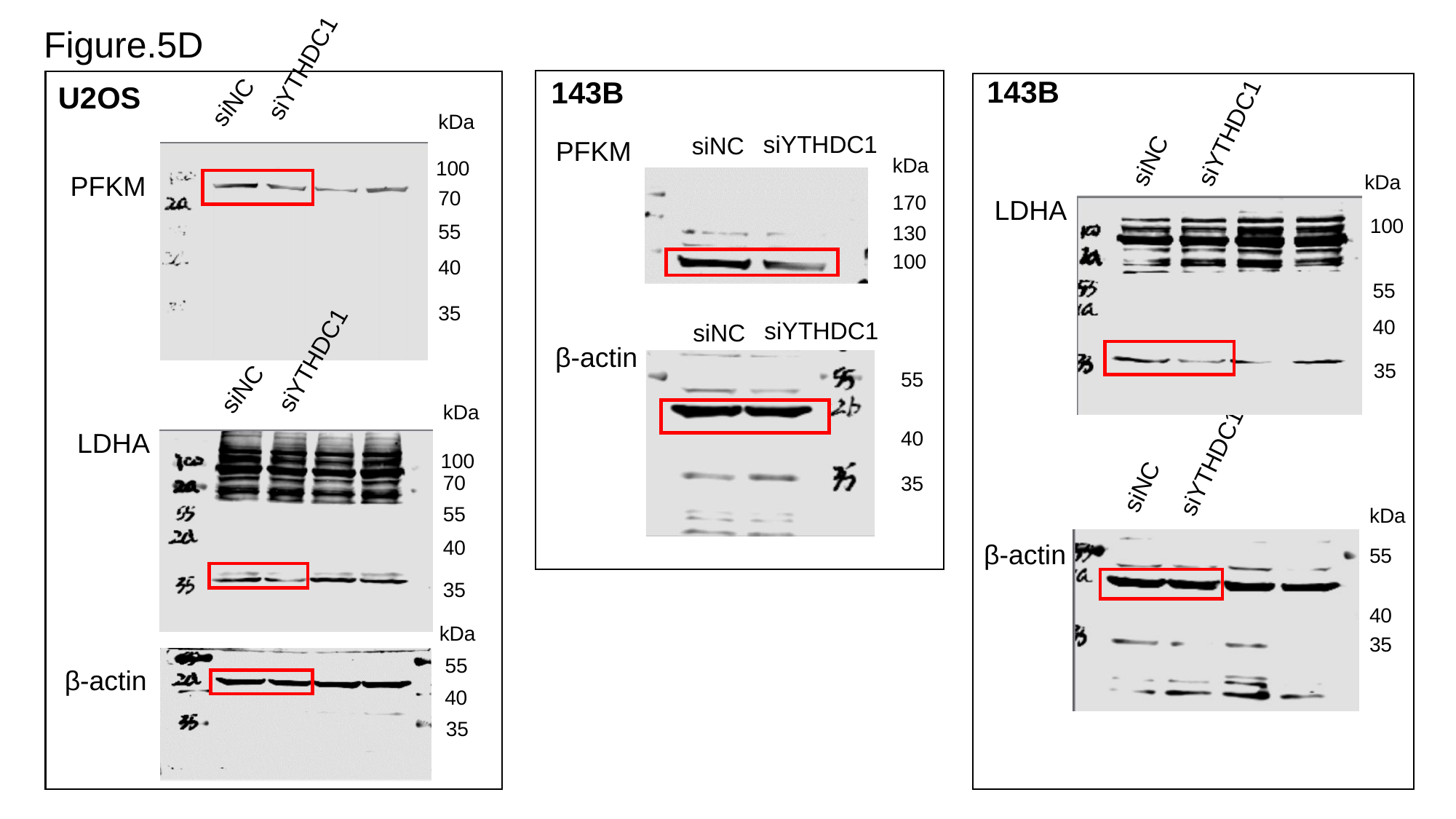

siYTHDC1
U2OS
siNC
kDa
100
PFKM
70
55
40
35
Figure.5D
siYTHDC1
siNC
kDa
LDHA
55
40
35
100
143B
143B
siYTHDC1
siNC
PFKM
kDa
170
130
100
siYTHDC1
siNC
kDa
LDHA
100
70
55
40
35
siYTHDC1
siNC
β-actin
55
siYTHDC1
siNC
kDa
β-actin
55
40
35
40
35
kDa
55
β-actin
40
35

## Slide 9
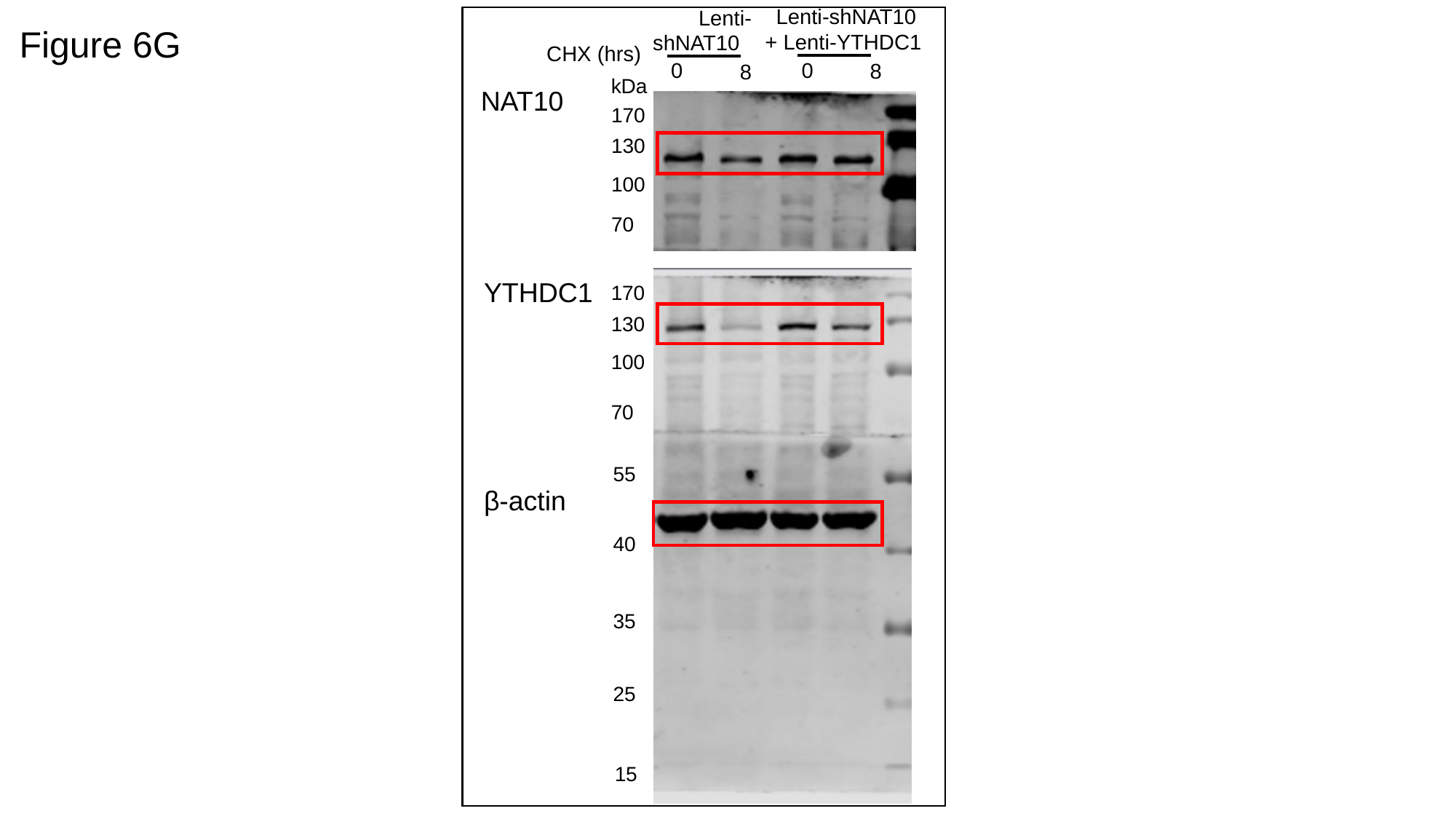

Lenti-shNAT10
+ Lenti-YTHDC1
Lenti-
shNAT10
kDa
NAT10
170
130
100
70
YTHDC1
170
130
100
70
55
β-actin
40
35
25
15
CHX (hrs)
0
8
0
8
Figure 6G

## Slide 10
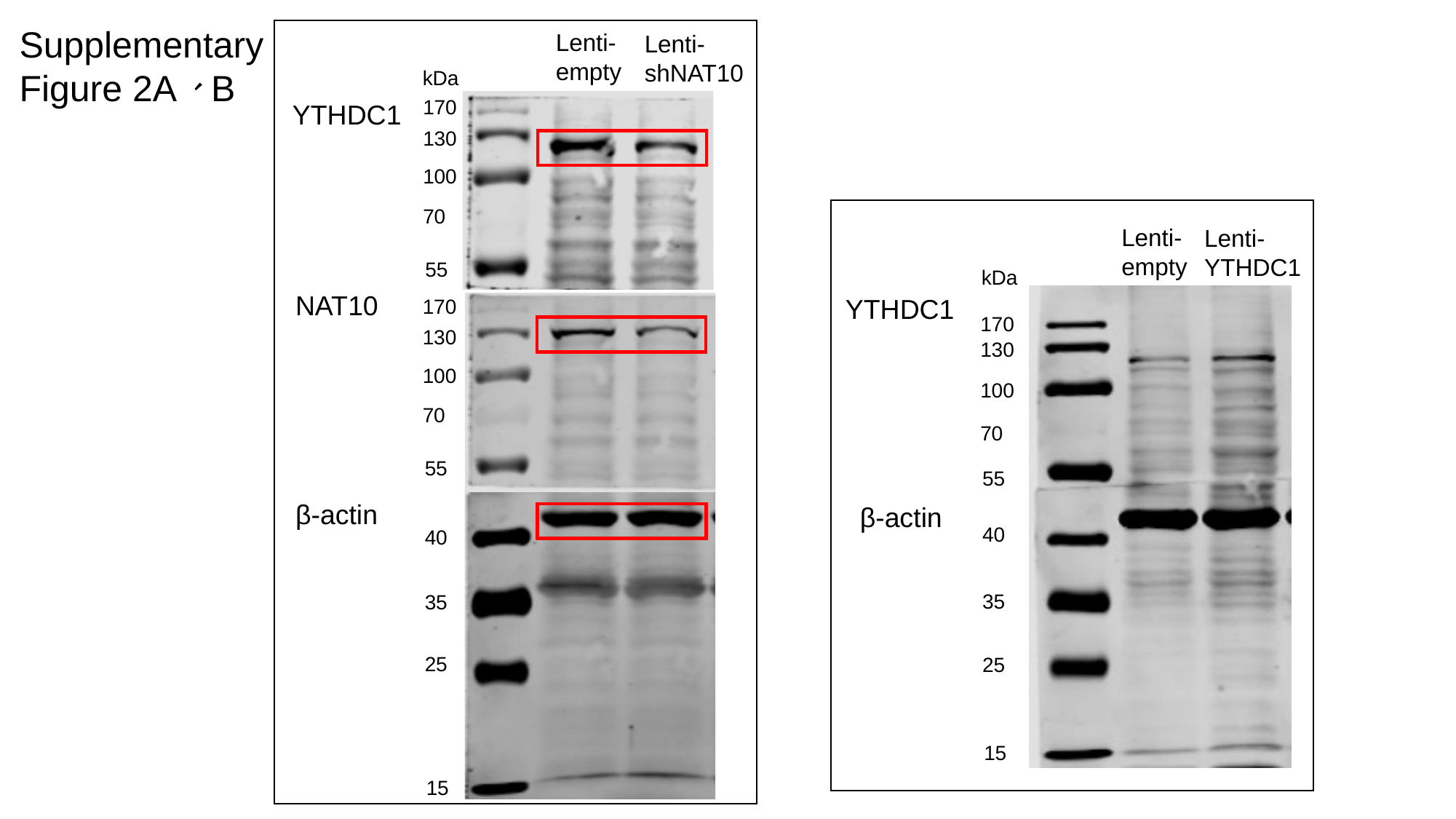

Supplementary Figure 2A、B
Lenti-empty
Lenti-shNAT10
kDa
170
YTHDC1
130
100
70
55
NAT10
170
130
100
70
55
β-actin
40
35
25
15
Lenti-empty
Lenti-YTHDC1
kDa
YTHDC1
170
130
100
70
55
β-actin
40
35
25
15
